# Influence of the Type of Nanofillers on the Properties of Composites Used in Dentistry and 3D Printing

**DOI:** 10.3390/ijms241310549

**Published:** 2023-06-23

**Authors:** Małgorzata Noworyta, Monika Topa-Skwarczyńska, Paweł Jamróz, Dawid Oksiuta, Małgorzata Tyszka-Czochara, Klaudia Trembecka-Wójciga, Joanna Ortyl

**Affiliations:** 1 Faculty of Chemical Engineering and Technology, Cracow University of Technology, Warszawska 24, 31-155 Cracow, Poland; malgorzata.noworyta@student.pk.edu.pl (M.N.);; 2 Faculty of Mechanical Engineering, Cracow University of Technology, Jana Pawła II 37, 31-864 Cracow, Poland; 3 Faculty of Pharmacy, Jagiellonian University Medical College, Medyczna 9, 30-688 Kraków, Poland; 4 Institute of Metallurgy and Materials Science, Polish Academy of Sciences, Reymonta 25, 30-059 Cracow, Poland; k.trembecka@imim.pl; 5 Photo4Chem Ltd., Lea 114, 30-133 Cracow, Poland; 6 Photo HiTech Ltd., Bobrzyńskiego 14, 30-348 Cracow, Poland

**Keywords:** nanomaterials, nanotechnology, photopolymerization, polymer nanocomposites, radical polymerization, cationic polymerization

## Abstract

Photopolymerization is a growing field with an extensive range of applications and is environmentally friendly owing to its energy-efficient nature. Such light-assisted curing methods were initially used to cure the coatings. However, it has become common to use photopolymerization to produce 3D objects, such as bridges or dental crowns, as well as to cure dental fillings. In this study, polymer nanocomposites containing inorganic nanofillers (such as zinc nano-oxide and zinc nano-oxide doped with two wt.% aluminum, titanium nano-oxide, kaolin nanoclay, zirconium nano-oxide, aluminum nano-oxide, and silicon nano-oxide) were fabricated and studied using Real Time FT-IR to investigate the effects of these nanoadditives on the final conversion rates of the obtained nanocomposites. The effects of the fillers on the viscosity of the produced nanocomposites were also investigated, and 3D prints of the selected nanocomposites were presented.

## 1. Introduction

Photopolymerization is a method of polymerization in which monomers or prepolymers are converted into polymers using light radiation, usually of a specific UV or visible (Vis) wavelength [1,2,3,4,5,6,7,8]. During photopolymerization, the photoinitiator added to the reaction system is decomposed by means of the absorption of light energy. As a result of this process, the resulting free radicals or radical ions activate the monomers, which then react with each other to form chemical bonds, leading to the formation of a three-dimensional polymer network. Photopolymerization is currently a growing field with a wide range of applications [8,9,10,11] and is considered an environmentally friendly curing method owing to its energy-efficient nature. Initially used mainly for curing coatings, it is now widely used in photocurable inks, varnishes, and adhesives, as well as in the fabrication of 3D objects, such as bridges and dental crowns, as well as for curing dental fillings [12,13,14,15,16,17,18,19,20].

Photopolymerization offers several benefits as a polymerization method. First, photopolymerization is an energy-efficient process [21,22,23]. This is a highly energy-efficient process compared to other polymerization methods because it requires only a short exposure to light to trigger the polymerization reaction, which can lead to reduced energy consumption and lower production costs. The second benefit is that it is a rapid process, which allows for the quick curing of materials. The polymerization reaction can be easily controlled by adjusting the intensity and duration of light exposure, providing precise control over the curing process and the resulting properties of the polymerized material. Moreover, photopolymerization can be initiated selectively in specific areas or regions. This phenomenon makes it suitable for applications in 3D printing [24,25,26,27,28,29,30,31,32,33,34,35], microfabrication [36,37,38,39], and photolithography [40,41]. The essential point is that photopolymerization can be used with various monomers and polymers, producing materials with tailored properties [2]. Processes initiated using light are additionally environmentally friendly because they do not generate harmful byproducts or emissions. The process does not require heat or solvents, and this can reduce its environmental impact compared to other curing methods. Nowadays, photopolymerization must be a “green” process, and it typically generates minimal waste. The new monomers can be quickly recovered and reused, thereby reducing material waste, in addition to promoting sustainability. Overall, photopolymerization offers many benefits, including energy efficiency, rapid curing, precise control, versatility, environmental friendliness, and reduced waste generation, making it a promising and widely used method across various applications.

Photopolymerization is widely used in 3D printing applications, particularly for the fabrication of nanocomposite materials [42,43,44,45,46,47,48,49,50,51]. Nanocomposites consist of a polymer matrix infused with nanoscale fillers, which can provide enhanced properties compared with traditional polymer materials [52,53]. Photopolymerization is used to cure a liquid resin containing a polymer matrix and nanofillers, converting it into a solid object, layer by layer. In this study, we developed a photopolymerization process for the 3D printing of nanocomposites with different nanoadditives. Using photocurable polymer nanocomposites in 3D printing allows for precise fabrication of dental restorations, such as dental crowns, bridges, dentures, and orthodontic appliances. The nanoscale fillers incorporated into the polymer matrix can vary in composition, size, and shape depending on the specific application requirements [54,55]. Common types of nanofillers used in dental nanocomposites include nanoparticles of silica, hydroxyapatite [43], and titanium dioxide [44,45], among others. These fillers can impart desirable properties onto printed dental restorations, such as improved mechanical strength, wear resistance, and biocompatibility. The photocurable nature of polymer nanocomposites allows layer-by-layer dental restoration fabrication using stereolithography or digital light processing (DLP). In this process, a digital dental restoration model selectively cures the polymer nanocomposite material layer-by-layer using a light source, typically UV or visible light [54,56]. The cured material then solidifies and forms the desired shape for dental restorations. This layer-by-layer approach enables the fabrication of complex geometries with high precision and accuracy, which is crucial for dental applications where customized restorations are often required [57]. The use of photocurable polymer nanocomposites in 3D printing for dental applications offers several advantages over traditional fabrication methods. These include reduced material waste, improved design flexibility, shorter production times, and enhanced mechanical properties in the final dental restorations [58,59]. Additionally, the biocompatibility of polymer nanocomposites can be tailored to suit the specific requirements of dental applications, ensuring that the fabricated restorations are safe for use in the oral environment [60]. Overall, photocurable polymer nanocomposites have emerged as promising materials for 3D printing in dental applications, offering numerous advantages in terms of fabrication efficiency, customization, and material properties.

This paper presents the results of a study on new cationic nanocomposites with potential dental applications, in addition to investigating the effects of the selected nanoadditives on the conversion rates and viscosity of monomers that are commonly used in dentistry. The polymerization of the composites was performed using the real-time FT-IR method, a D-Light PRO dental lamp, and a 3D-DLP printer. The motivation for this work was the fact that nanomaterials offer several advantages, including the ability to fabricate complex and customized objects, precise control over the curing process, and incorporate nanofillers in order to achieve such enhanced properties as improved mechanical strength, thermal stability, and electrical conductivity. This makes it suitable for various applications, including aerospace, the automotive industry, electronics, healthcare, and consumer goods, as they require materials with advanced properties.

## 2. Experimental

### 2.1. Materials

**Figure 1:**
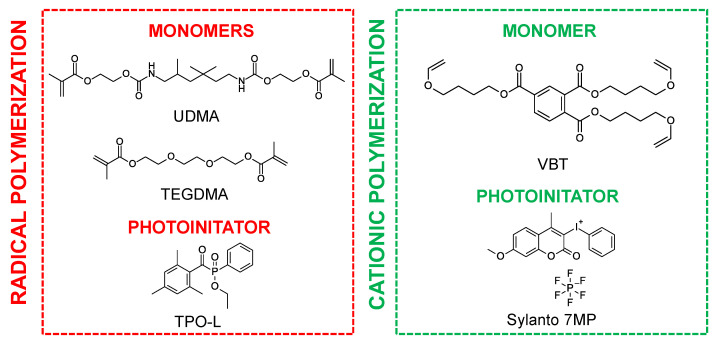
Chemical structures of monomers and photoinitiators.

A mixture of urethane dimethacrylate (UDMA, Sigma Aldrich, Burlington, MA, USA) and triethylene glycol dimethacrylate (TEGDMA, Sigma Aldrich) in a weight ratio of 7:3 was used as the organic matrix for cationic photopolymerization, while the trifunctional vinyl monomer tris [4-(vinyloxy)butyl]trimelliate (VBT, Sigma Aldrich) was used as the organic matrix for cationic photopolymerization. The well-known nontoxic phosphine initiator 2,4,6-trimethylbenzoylphenylphosphonic acid ethyl ester (TPO-L, Angene, London, UK) was used as a radical initiator, whereas the initiator for initiating the cationic polymerization of the VBT monomer was Sylanto 7 MP. The chemical structures of the components are shown in Figure 1. 

The following particles were used to increase the strength of the composition:

− ZnO, with a size of 10–30 nm (US Research Nanomaterials, Inc., Houston, TX, USA);
− ZnO oxide nanoparticles doped with 2 wt.% of pure aluminum (i.e., AlZnO), with size of 15 nm (US Research Nanomaterials, Inc.);
− Kaolin clay nanoparticles, Al_2_Si_2_O_5_(OH)_4_–2 H_2_O, with a diameter of 30–70 nm and length of 1–3 µm (Sigma Aldrich);
− titanium oxide nanoparticles, TiO_2_, with a size of less than 25 nm (Sigma Aldrich)
− Aluminum oxide nanoparticles, Al_2_O_3_, with a size of approximately 13 nm (Sigma Aldrich)
− Silicon oxide nanoparticles, SiO_2_, 10–20 nm (Sigma Aldrich)
− Zirconium oxide nanoparticles, ZrO_2_, with a size of less than 100 nm (Sigma Aldrich).

### 2.2. Absorbance Measurement

A Silver Nova spectrometer and a tungsten-deuterium lamp (StellarNet Inc., Tampa, FL, USA) were used to perform the spectrophotometric studies. The absorbance spectra of solutions placed in a quartz cuvette with an optical path of exactly 1 cm were recorded. The solutions used for the measurements were a TPO-L photoinitiator solution of 12.58 $\times \;10^{-4}$ mol/dm^3^, and a Sylanto 7 MP photoinitiator solution of $4.05\;\times \;10^{-5}$ mol/dm^3^.

### 2.3. Photostability Measurement

Photodecomposition studies of the photoinitiators were carried out using a SilverNova spectrometer and a broadband deuterium-tungsten UV–Vis lamp. Vis-LEDs at 405 nm L3 (Thorlabs Inc., Newton, NJ, USA) were used to expose the prepared solutions. Measurements were carried out for 20 min in a quartz cuvette with four translucent walls with an optical path of 1 cm, containing 3 mL of the analyzed solution and using a current power of 870 mW/cm^2^, corresponding to 1000 mA. A DC2200 (Thorlabs Inc.) was used as the power supply for the diodes. For the measurements, a solution of the photoinitiator TPO-L in acetonitrile was used, with a concentration of 12.58$\;\times 10^{-4}$ mol/dm^3^, in addition to Sylanto 7 MP in acetonitrile, with a concentration of 4.05$\;\times 10^{-5}$ mol/dm^3^.

### 2.4. Real-Time FT-IR Photopolymerization Measurements

The kinetics and conversion rates of the polymer compositions were measured in real time using Fourier-transform infrared spectroscopy. The equipment used was a Thermo Scientific i10 NicoletTM spectrometer, which had a special horizontal attachment that allowed for online photopolymerization. A 405 nm L4 Vis-LED from Thorlabs Inc. was used to irradiate the samples during the measurements, with a sample intensity of up to 1000 mW/cm^2^. For radical photopolymerization processes, a Vis-LED (with an emission maximum at 405 nm and a sample intensity of 75 mW/cm^2^) was used; for radical polymerization, the same diode (with a sample intensity of 500 mW/cm^2^) was used. An optical fiber measuring 1.2 m in length and 0.5 cm in diameter was responsible for delivering light radiation to the sample. Illumination of the tested nanocompositions was started 10 s after the start of the measurement data recording. OMNIC software was used to record measurement data. Measurements were taken for compositions with nanocompositions containing 1 wt.% and 5 wt.% nanofillers for layer thicknesses of 25 μm and 1.4 mm. The degree of conversion was calculated using Equation (1):

(1) $$Conversion\;\left[\%\right]=\left(1-\frac{A}{A_{0}}\right)*100\;\%$$

where *A* is the area of the monitored band, and *A*_0_ is the initial value of the area of the monitored band.

### 2.5. Viscosity Measurements

The viscosity of the fabricated polymer nanocomposites was measured using an MCR302e Rheometer (Anton Paar, Ashland, VA, USA) The test was conducted at a constant temperature of 25 °C, maintained using a thermostat. The measurement gap between the PP25 SN73155 spindle and platform was 0.1 millimeter. The measurement in the RheoCompass software was set to a variable head speed (shear rate) ranging from 1/s to 100/s, and the measurements were performed for 550 s. The head movement profile was set to Low Viscosity. Measurements were taken for compositions containing all nanofillers at 1 wt.% or 0.1 wt.% and 5 wt.% or 0.5 wt.% for compositions dedicated to cationic and radical polymerization, as well as 10 wt.% and 50 wt.% of selected compositions with nanofillers (titanium nano-oxide, kaolin nanoclay, zirconium nano-oxide, aluminum nano-oxide, and silicon nano-oxide at only 10 wt.%).

### 2.6. Curing Compositions Using the Dental Lamp

A two-wavelength D-Light PRO diode dental lamp (GC) for photocuring materials was used to cure the compositions containing 10 wt.% and 50 wt.% nanoadditives. The nanocomposition was cured in the high-power mode of the lamp, i.e., 1400 mW/cm^2^ so-called HP (light in the wavelength range 400 to 480 nm). A 20 s exposure was repeated three times for each sample, i.e., a total exposure time of 1 min for the composite. The actual lamp power measured with an optical meter was 493 mW/cm^2^.

### 2.7. 3D printing of Nanocomposites

**Figure 2:**
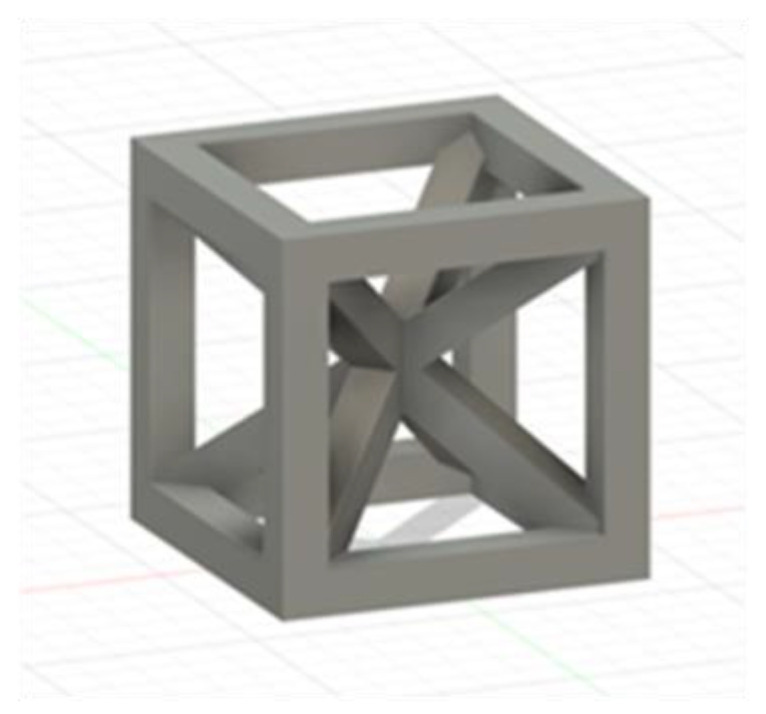
Cube design made in Fusion software.

An Anycubic printer, the Photon Mono X, was used to make prints from a radical polymerizing nanocomposition with 5% by weight of nanofillers. The light intensity, measured using a PM160 optical meter (Thorlabs Inc.), was 15.63 mW/cm^2^. A 10 × 10 mm cube (Figure 2), designed using Fusion software, was subjected to the printing process. The printing of each composition was performed with irradiation of the first three layers for 10 s, and each subsequent layer for 6.5 s. A DSX1000 digital microscope (Olympus, Miami, FL, USA) was used to obtain images.

### 2.8. Surface Analysis of 3D Prints

#### 2.8.1. Noncontact 3D Surface Texture Metrology

**Figure 3:**
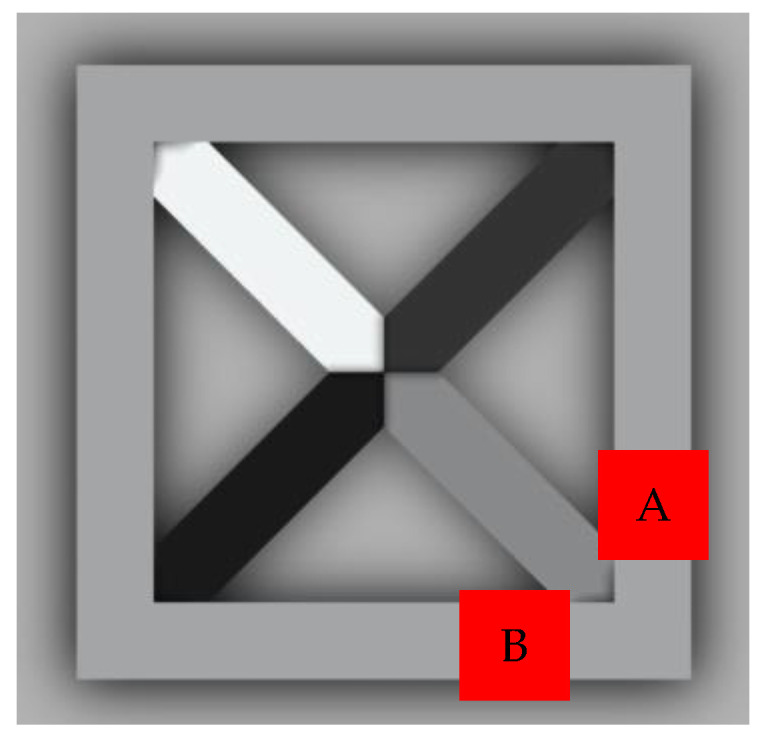
Top view of the 3D print showing the locations of measurements A or B.

The ContourX-200 Optical Profilometer (Bruker, Billerica, MA, USA), which could provide a vertical resolution of less than 0.01 nm and horizontal resolution of up to 0.15 µm, was used to examine the surface roughness and waviness of the prints. The device provided a measurement speed of 37 µm/s. During the measurement, the WLI (White Light Interferometry) method and a Mirau 20X objective were used. Prints polymerizing according to the radical mechanism with 5% by weight of nanoadditives were tested. The measurement area for each sample was one of the points marked in Figure 3.

#### 2.8.2. SEM Research of Selected 3D Prints

A Philips/FEI ESEM-XL30 equipment in SE mode was used for the SEM (Scanning Electron Microscopy) investigations, which required an accelerating voltage of 10 kV. A thin layer of carbon was applied onto the samples before examination. Prints polymerized using a radical mechanism containing 5% by weight zirconium dioxide, 5% by weight kaolin nanoclay, in addition to a base sample consisting of TPO-L photoinitiator and a mixture of UDMA/TEGDMA monomers, were subjected to SEM examination.

## 3. Results

### 3.1. Spectroscopic Measurements

**Figure 4:**
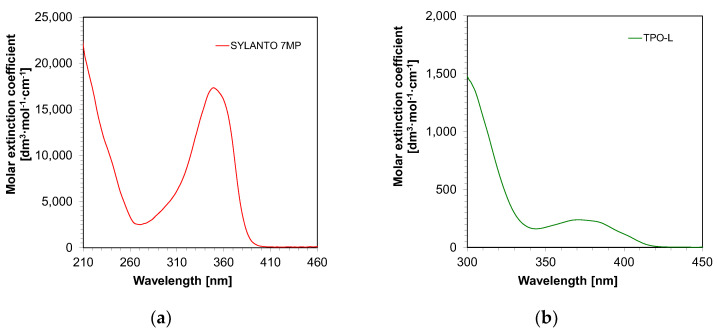
Absorption spectrum of (**a**) Sylanto 7 MP photoinitiator dedicated to cationic photopolymerization, and (**b**) TPO-L photoinitiator dedicated to radical photopolymerization.

First, the absorbance measurements of the initiators in acetonitrile were performed. It was demonstrated that both TPO-L and Sylanto 7 MP absorbed in the range up to just over 400 nm (Figure 4). For the TPO-L photoinitiator, the wavelength for the maximum molar extinction coefficient was shifted towards longer wavelengths, compared to the Sylanto 7 MP photoinitiator. The maximum molar extinction coefficient at the longest wavelength band (349 nm) for the photoinitiator Sylanto 7 MP was 17,347 dm^3^·mol^−1^·cm^−1^ (Table 1). The maximum molar extinction coefficient in the longest wavelength band at 371 nm for the TPO-L initiator was 239 dm^3^·mol^−1^·cm^−1^. 

However, the molar extinction coefficient at 405 nm was 83 dm^3^·mol^−1^·cm^−1^ for the TPO-L initiator, and 105 dm^3^·mol^−1^·cm^−1^ for the Sylanto 7 MP initiator, which suggested that these compounds were suitable for initiating photopolymerization processes using light sources with emission maxima at 405 nm. This is extremely important, as LEDs with maximum emission at that wavelength are commonly used in both 3D printers and polymerization lamps within the dental industry.

### 3.2. Photolysis Measurements

**Figure 5:**
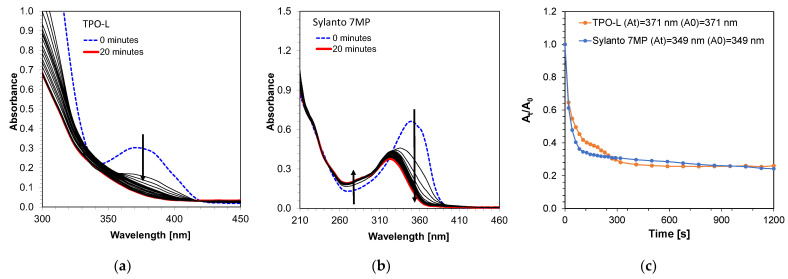
Photodecomposition of photoinitiator (**a**) TPO-L (12.62 · 10^−4^ mol·dm^−3^); (**b**) Sylanto 7 MP (4.05 · 10^−5^ mol·dm^−3^) at 20 min and intensity at 870 mW/cm^2^ on the sample; (**c**) Time dependence of absorbance intensity quotient at wavelength for Sylanto 7 MP at 349 nm and TPO-L at 371 nm on initial absorbance intensity at these wavelengths over time.

Photostability measurements of these initiators were also performed using a diode with an emission maximum at a wavelength of 405 nm and a sample intensity of 870 mW/cm^2^ for 20 min (Figure 5). The TPO-L photo decay occurred significantly in the first few seconds of exposure of the system (Figure 5a). The most intense decay occurred up to approximately 150 s, suggesting that the compound subsequently decayed into the active radicals responsible for initiating the photopolymerization process. In addition, photobleaching of the compound was observed under Vis-LED irradiation. The decomposition of the Sylanto 7 MP initiator (Figure 5b) under the same conditions as cationic radicals also occurred rapidly, with an additional shift in the absorption band towards shorter wavelengths during photolysis. The decomposition of the Sylanto 7 MP initiator occurred comparably to that of TPO-L (Figure 5c).

### 3.3. Viscosity of Nanocomposites

The viscosity of composites is extremely important in both 3D printing processes and composites in dentistry. In the case of 3D printing, the viscosity of the composite determines the resolution of the print, whereas in the case of dental composites, viscosity affects the application process of dental fillings. 

Therefore, in the next step, the viscosities of the developed compositions were measured. The base compositions of the nanocomposites were, in the case of the cationic photopolymerization mechanism, a mixture of VBT monomer with Sylanto 7 MP initiator 1% by weight, whereas in the case of radical photopolymerization, it was a mixture of UDMA/TEGDMA monomers in a weight ratio of 7:3 and TPO-L photoinitiator 1% by weight. Appropriate amounts of inorganic fillers were added to the base compositions (Figure 6). The shear-rate dependence of the viscosity of the compositions was determined (Figures S1–S27).

**Figure 6:**
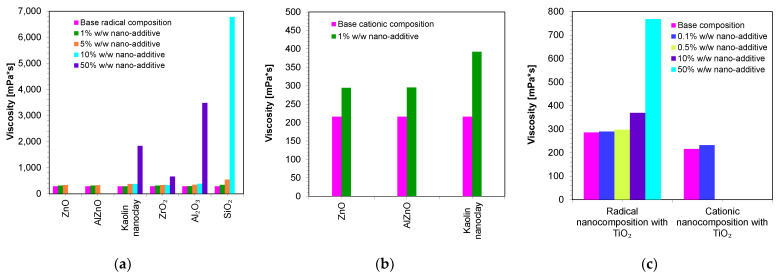
Comparison of the viscosity of nanocompositions containing different concentrations of nanoadditives: (**a**) polymerizing with a radical mechanism and (**b**) cationic mechanism; (**c**) comparison of viscosity for compositions being polymerized with the cationic and radical mechanisms and additionally containing titanium nano-oxide.

Figure 6 presents a comparison of the viscosities of the nanocomposites containing different amounts of nanofillers. It was then demonstrated that the base composition (without nanofillers) had low viscosity. However, it was only the addition of nanofillers that increased the viscosity of the polymerizing composites according to the radical mechanism (Figure 6a), as well as according to the cationic mechanism (Figure 6b). In addition, it was proven that the use of different fillers with the same amount (up to 5 wt.%) did not significantly influence the final viscosity values. However, it is interesting to note that even with the addition of 10 wt.% of SiO_2_ to the base composition, polymerizing according to the radical mechanism increased the viscosity to nearly 6781 mPa·s, suggesting that silica causes the highest increase in the viscosity of the composition. It was also observed that the type of the organic matrix affected the final viscosity. It was therefore clearly demonstrated that a composition polymerized according to the cationic mechanism had a slightly lower viscosity value compared to compositions polymerized according to the radical mechanism. This was due to the fact that the VBT monomer had a low viscosity at room temperature (216 mPa·s) (Table 2). However, it is worth noting that UDMA/TEGDMA monomer mixtures are widely used in the production of dental composites.

### 3.4. Real-Time FT-IR Studies of Nanocompositions

**Figure 7:**
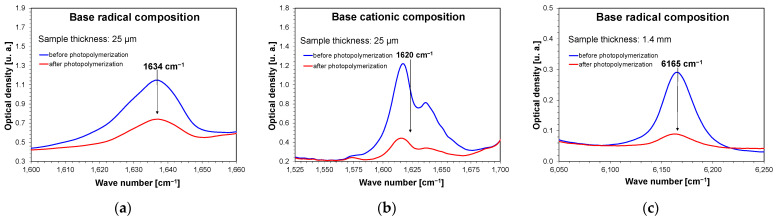
Changes in FT-IR spectra demonstrating band decay at (**a**) wave number 1634 cm^−1^ for thin film compositions polymerizing with a radical mechanism; (**b**) wave number 1620 cm^−1^ for thin film compositions polymerizing with a cationic mechanism; (**c**) wave number 6165 cm^−1^ for 1.4 mm thick compositions polymerizing with a radical mechanism.

The photopolymerization process was then analyzed using real-time FT-IR. All the base compositions and compositions containing 1 wt.% and 5 wt.% nanofillers were independently subjected to the process. An exception to this was titanium oxide, which had compositions of 0.1 wt.% and 0.5 wt.% of this filler tested. To determine the influence of the selected inorganic nanofillers on the radical and cationic photopolymerization process, the nanocomposites were irradiated with a 405 nm Vis-LED with a light intensity of 75 mA (1.5 mW/cm^2^) on the sample for 480 s for radical nanocompositions, and with a light intensity of 500 mA (10 mW/cm^2^) for 900 s for cationic nanocompositions. The diode for each measurement was switched on 10 s after the start of spectrum recording. The exact compositions of the mixtures are presented in the Experimental section. Real-time Fourier transform infrared (FT-IR) measurements were performed during irradiation. During photopolymerization, the acrylate groups disappeared for the UDMA and TEGDMA monomers and vinyl groups for the VBT monomer. During the photopolymerization of the UDMA/TEGDMA monomers, band loss was observed at a wavenumber of 1634 cm^−1^ (Figure 7a and Figures S40–S53), and for the VBT monomer, band loss was observed at a wavenumber of 1620 cm^−1^ (Figure 7b and Figures S54–S57). In the case of carrying out measurements for thick films (1.4 mm), the band at 1634 cm^−1^ was completely saturated, and it was impossible to monitor the disappearance of this band; therefore, in this case, for both cationic and radical compositions containing nanofillers, the band was monitored at the wave number of 6165 cm^−1^ (Figure 7c and Figures S58–S76). The conversion rates of the studied compositions were obtained by monitoring the real-time change in the peak area at a given wavenumber. Equation (1) was used for these calculations.

#### 3.4.1. Thin Film Compositions (25 µm) Polymerizing with a Radical Mechanism

**Figure 8:**
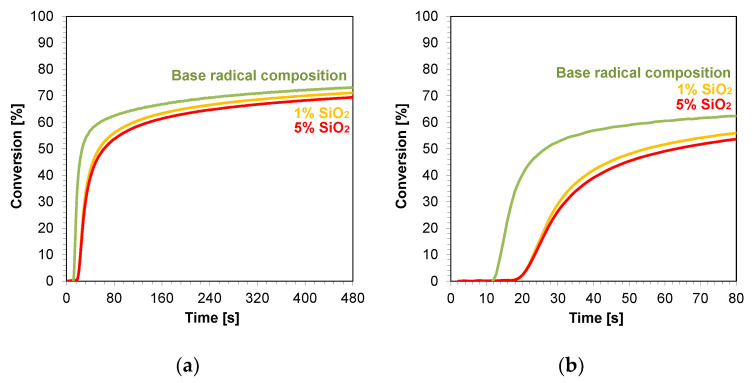
Kinetic profiles demonstrating the photopolymerization process of nanocompositions reacting according to a radical mechanism containing different weight concentrations of silicon nano-oxide for (**a**) 480 s, and (**b**) 80 s.

The kinetic profiles obtained using the photopolymerization from nanocompositions according to the radical mechanism for 25 μm thick samples were characterized by a large scatter of induction times, which ranged from 11 to 21 s (Table 3). The smallest induction time was obtained for the base composition, while the longest induction time was characterized using nanocompositions containing silicon nano-oxide (21 s) (Figure 8a,b). A significant increase in the induction time was also observed for the use of the mixture with aluminum oxide (Figure S33) as a filler and, to a lesser extent, the use of nanometric ZrO_2_ (Figure S32). The addition of 5 wt.% of the aforementioned kaolin nanoclay also increased the induction time of the nanocomposition relative to the base composition. For nanocomposites with fillers, such as pure zinc oxide, zinc oxide doped with 2 wt.% aluminum, titanium oxide, and 1 wt.% kaolin nanoclay, induction times similar to those of the base composites were recorded (Figures S28–S31). Using ZnO, AlZnO, TiO_2_, and a 5 wt.% kaolin nanoclay and 5 wt.% ZrO_2_ as nanofiller, the final conversion rate of the nanocomposites relative to the base composition was improved. The nanocomposite with 5 wt.% AlZnO showed the highest final conversion rate of 79%, compared to the radical base composition, for which the conversion rate was 73%. The introduction of the other nanocomposites resulted in a slight decrease in the conversion of the nanocomposite relative to the base composition by up to 3%.

In conclusion, the addition of nanofillers to the base composition affected the photopolymerization rate, and the introduction of nanofillers decreased the photopolymerization rate (Figure 8). Nevertheless, the introduction of fillers is important in the production of composites, including dental composites, because they improve their mechanical properties.

#### 3.4.2. Thin Film Compositions (25 µm) Polymerizing with a Cationic Mechanism

The cationic photopolymerization process in the thin films was carried out in a manner analogous to the radical photopolymerization process. However, in this measurement, we used a Vis-LED with an emission maximum at a wavelength of 405 nm and a higher intensity on the sample of 500 mA. This was due to the fact that the Sylanto 7 MP initiator was less efficient than the TPO-L initiator and, in addition, polymer compositions that initiate using a radical mechanism generally polymerized with greater efficiency. 

**Figure 9:**
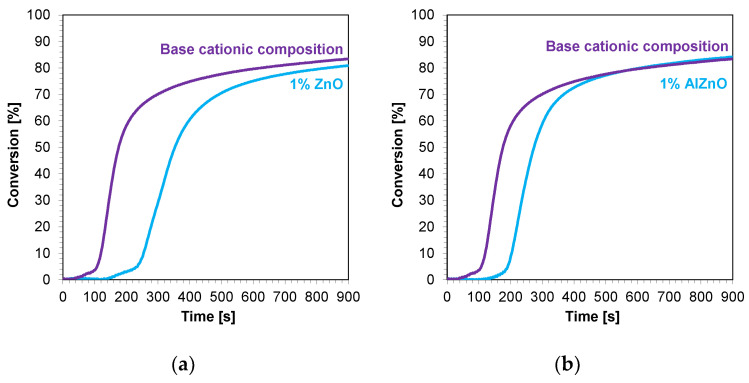

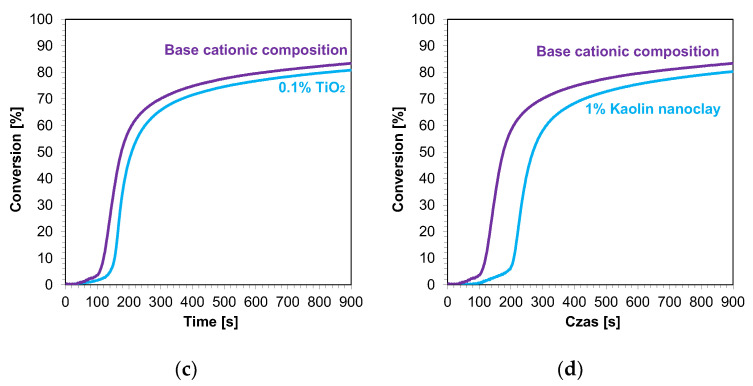
Kinetic profiles demonstrating the photopolymerization process of nanocompositions reacting according to a cationic mechanism containing different weight concentrations of (**a**) zinc nano-oxide; (**b**) zinc nano-oxide doped with 2 wt.% aluminum; (**c**) titanium nano-oxide; (**d**) kaolin nanoclay, and a layer thickness of 25 μm.

The kinetic profiles of the base compositions with the addition of 1 wt.% nanofillers are shown in Figure 9a–d. In each case, the introduction of 1 wt.% nanofiller into the base composition increased the induction time and slowed the rate of cationic photopolymerization. This indicates that these fillers impeded the penetration of light into the composites. Nevertheless, the final conversion rates for the blends tested were high, ranging from 79% to 85% (Table 4).

**Figure 10:**
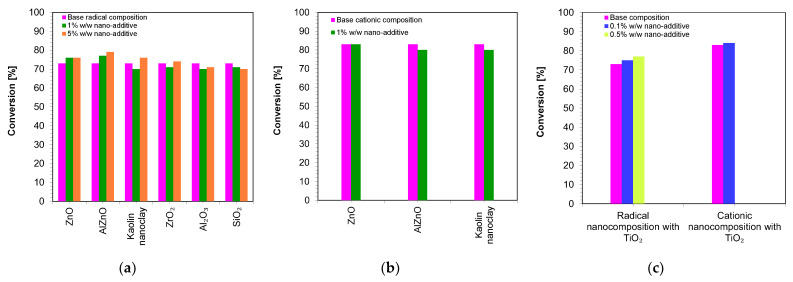
A comparison of the final conversion degrees for compositions: (**a**) polymerizing according to the radical mechanism; (**b**) polymerizing according to the cationic mechanism; (**c**) comparison of the final conversion degrees of a composition polymerizing according to the cationic and radical mechanism containing a TiO_2_ nanocomposite (The thickness of the composition was 25 µm).

Figure 10 shows a comparison of the final conversion degrees for compositions polymerizing according to the radical mechanism and cationic mechanism. It can clearly be seen that all of the studied compositions polymerized to a high degree, above 70%.

#### 3.4.3. Thick Film Compositions (1.4 mm) Polymerizing with a Radical Mechanism

**Figure 11:**
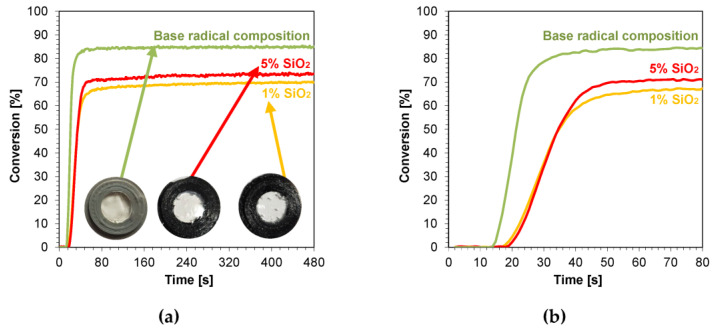
Kinetic profiles demonstrating the photopolymerization process of nanocompositions reacting according to a radical mechanism containing different weight concentrations of silicon nano-oxide for (**a**) 480 s, and (**b**) 80 s (the layer thickness was 1.4 mm).

Samples of nanocomposites with a thickness of 1.4 mm were also photopolymerized. This choice was dictated by the fact that composites with a thickness of approximately 1.4 mm are applied in layers in dental practice. An organoleptic color analysis of the samples after the curing process was performed, and photographs of the resulting nanocomposites are shown in Figures S34–S39. The scattered kinetic curves indicate band saturation; however, this did not significantly hinder the final conversion steps. The addition of nanoadditives such as ZnO, AlZnO, TiO_2_, ZrO_2_, and Al_2_O_3_ to the radical base composition resulted in a white coloration of the nanocomposite derived from the color of the nanopowders. Increasing the concentration of these nanoadditives had little to no effect on making the nanocomposite with a higher oxide content whiter than its 1 wt.% concentration. No change in transparency or color was observed for the silicon oxide composition (Figure 11a). The calculated induction times of the tested samples oscillated between 11 s for the nanocompositions containing AlZnO and 1 wt.% ZnO, and 23 s for the nanocomposition containing nanometric alumina. The induction time for the base mixture was 14 s. None of the produced nanocomposites achieved a better conversion rate than the base mixture containing the monomers and photoinitiator, whereas compositions with 0.1 wt.% titanium nano-oxide and kaolin nanoclay showed very similar conversions to the base composition, 85% and 84% respectively for both nanoclay concentrations. The amount of nanoadditives, such as zirconium oxide, alumina, and silica, significantly worsened the conversion scope relative to the base composition (Table 3).

#### 3.4.4. Thick Film Compositions (1.4 mm) Polymerizing with Cationic Mechanism

**Figure 12:**
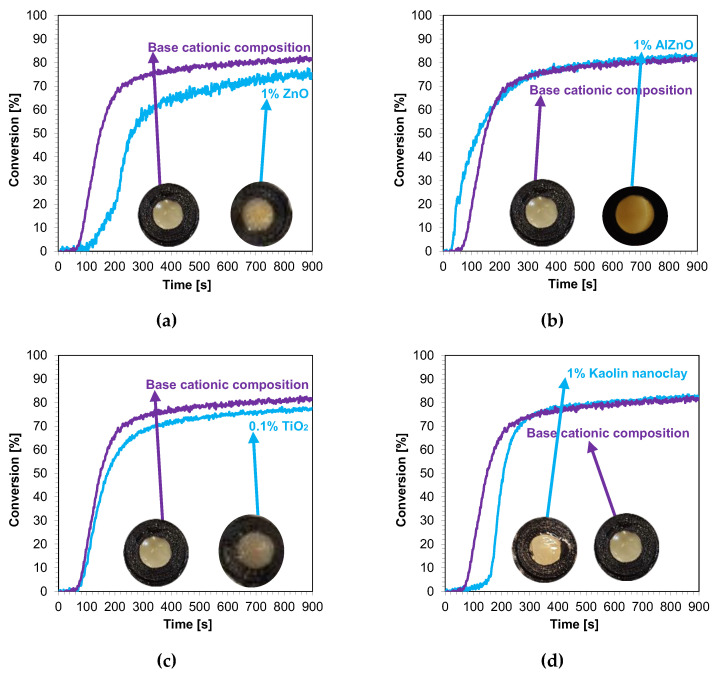
Kinetic profiles showing the photopolymerization process of the cationic base composition and containing 1 wt.% nanofiller: (**a**) zinc nano-oxide; (**b**) zinc nano-oxide doped with 2 wt.% aluminum; (**c**) titanium nano-oxide; (**d**) kaolin nanoclay (the layer thickness was 1.4 mm).

**Figure 13:**
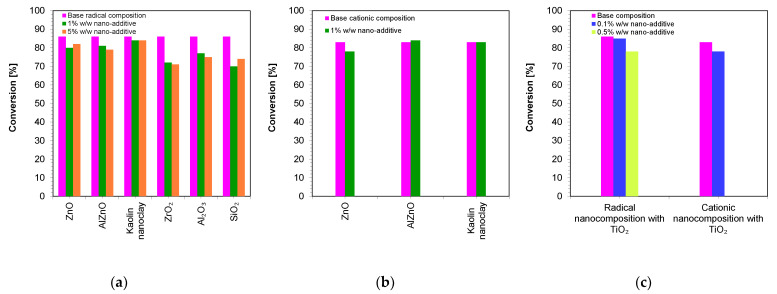
A comparison of the final conversion degrees for compositions: (**a**) polymerizing according to the radical mechanism; (**b**) polymerizing according to the cationic mechanism; (**c**) comparison of the final conversion degrees of a composition polymerizing according to the cationic and radical mechanism containing a TiO_2_ nanocomposite (The thickness of the composition was 1.4 mm).

In the next step, cationic photopolymerization was carried out in the so-called thick films for nanocomposites with 1 wt.% filler content. The cured nanocomposites were characterized by a flesh color derived from the color of the photoinitiator (Figure 12a–d). The addition of nanofillers, such as ZnO, AlZnO, or TiO_2_, resulted in the complete disappearance of the transparency of the obtained composites compared to the base composition (Figure 13). The nanoclay used as an additive in the composition caused a slight change in the color. The addition of nanoclay did not significantly affect the conversion rate of the composition compared to the composition polymerized according to the cationic mechanism without the addition of fillers; however, it did increase the induction time. The application of zinc oxide slowed down the entire photopolymerization process and caused a decrease in the degree of conversion of the nanocomposition in relation to the base composition. The titanium oxide in the system did not cause a significant change in the induction time, but it did adversely affect the degree of conversion. For the composition containing AlZnO in an amount of 1 wt.%, there was an increase in the rate of photopolymerization and a slight improvement in the final degree of conversion of the nanocomposition. The induction time for the cationic nanofiller systems ranged from 30 s for 1 wt.% AlZnO to as much as 158 s for 1 wt.% kaolin clay, and the conversion rates obtained in the process were high, ranging from 78% to 84% (Table 4).

A summary of the final conversion rates for the thick films is presented in Figure 12. As in the case of thin films, in thick films, the final conversion rate for all composites tested was greater than 70%, demonstrating that these composites have the potential to be used in dental composites and 3D printing.

### 3.5. Application Studies—Curing of Nanocomposites Using a Dental Lamp

**Figure 14:**
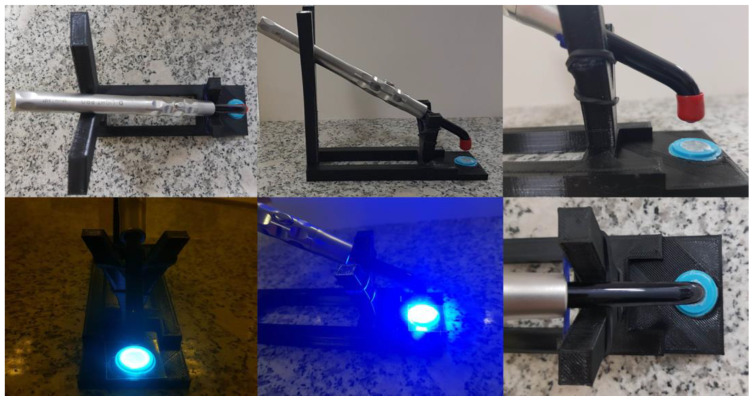
The stand for the photopolymerization process using a dental lamp.

To cure 1.4 mm thick radical nanocompositions containing 10 wt.% or 50 wt.% in nanoadditives (such as TiO_2_, kaolin nanoclay, Al_2_O_3_, ZrO_2_ or SiO_2)_, a stand was printed, on which a D-Light PRO dental lamp was placed (Figure 14). This allowed the lamp to be held at a constant distance from the sample and under conditions similar to those of real dental-filling application. The intensity of the incident light on the sample was 494 mW/cm^2^. The light source of the lamp was positioned over the specimen such that the incident light was evenly distributed over the surface of the light-curing composition.

Each cured sample was exposed thrice, for 20 s. Nanocompositions with a 10 wt.% and 50 wt.% titanium nano-oxide did not polymerize to any significant degree, and still remained liquid after the dental-lamp curing process. None of the other exposed mixtures had a visibly unpolymerized composition, but a sticky layer remained on the samples. In the case of the nanocomposition with 50 wt.% kaolin nanoclay, there was visible polymerization shrinkage. The addition of each nanofiller, with the exception of silicon oxide, reduced the transparency of the composite relative to the base sample. The resulting nanocomposites were distinctly white in color for titanium oxide and zirconium oxide. In the case of kaolin clay, the nanocomposite was milky white, and that with alumina was cloudy. During photopolymerization with the dental lamp, the colors of the individual nanocomposites before and after curing did not change significantly (Table 5).

### 3.6. 3D Printing Using Polymerizing Nanocomposites with a Radical Mechanism

The MSLA Anycubic Photon Mono X 3D printer, which operated based on the principle of exposure to light from a projector photocurable resins (DLP printing technology), was used to produce prints with 5 wt.% of nano zinc oxide, nano zinc oxide doped with 2 wt.% of aluminum, nano titanium oxide, kaolin nanoclay, nano zirconium oxide, nano aluminum oxide and nano silicon (Figures S77–S84). The exposure of the first three layers of the print was set to 20 s, and the light exposure for each subsequent layer was 6.5 s. Both microscopic and macroscopic camera images of the resulting prints were obtained. 

**Figure 15:**
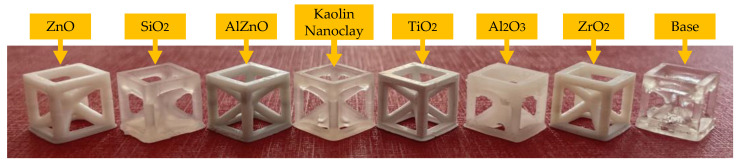
Summary of print images taken with the camera.

It was then shown that depending on the inorganic filler used, the print resolution and color were different (Figure 15).

**Figure 16:**
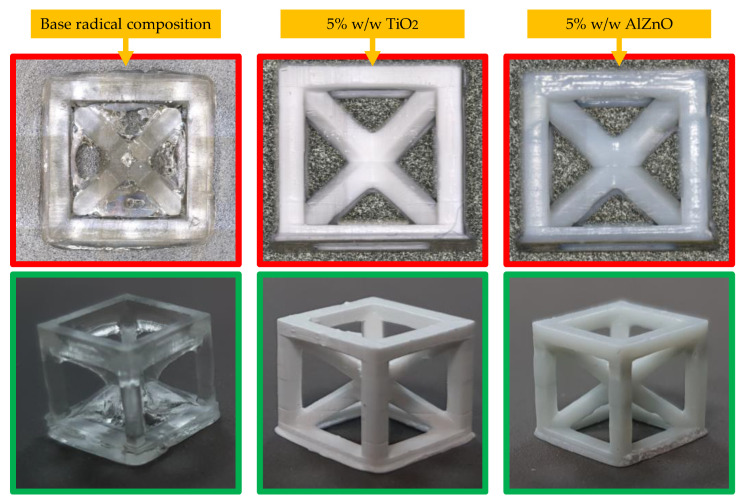
Photographs of example prints taken with an Olympus microscope (**top pictures**) and camera (**bottom pictures**).

The highest resolution was obtained for a composition containing 5 wt.% TiO_2_ and 5 wt.% AlZnO. Nevertheless, all the compositions had satisfactory resolution. A comparison of the best-printed cubes is shown in Figure 16.

### 3.7. Analysis of the Surface of 3D Prints Made with Polymerizing Nanocomposites According to Radical Mechanization

#### 3.7.1. Roughness and Waviness Tests Performed Using a Noncontact 3D Surface Texture Metrology Using the Optical Profilometer 

**Figure 17:**
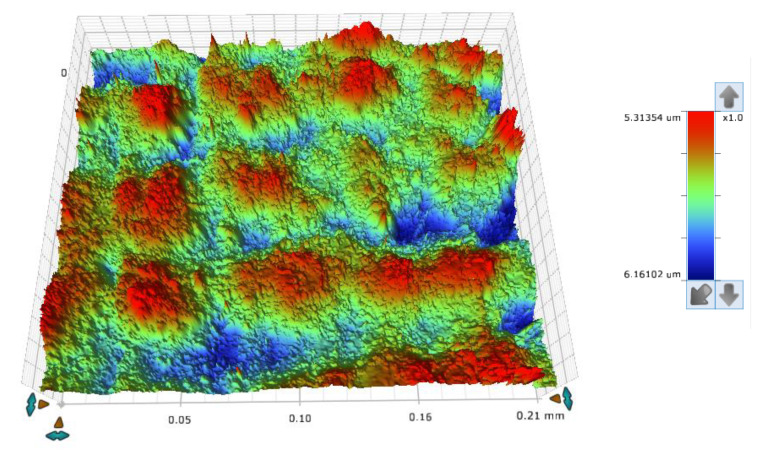
The surface topography of a 3D print made from a nanocomposition containing 5% by weight of silicon oxide.

**Figure 18:**
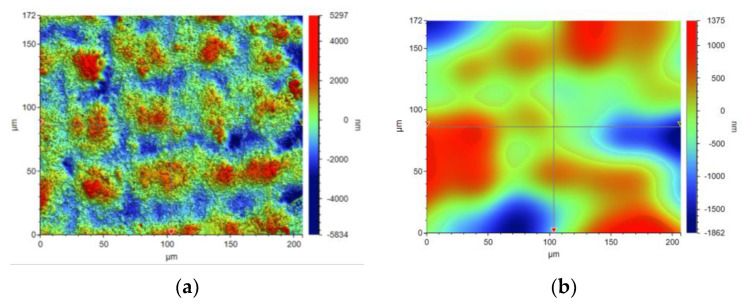
Visualization of the roughness (**a**) and waviness (**b**) of the test area of the print from the nanocomposition containing 5% by weight SiO_2_.

The concept of measuring surface roughness is essential for preventing uncertainty and disputes over the quality of the products. This parameter has become a common identifier, and is used throughout industry for validating manufacturing processes, confirming adherence to both internal and regulatory specifications, and guaranteeing the quality and performance of end products. The first important parameter developed for visualization was mean roughness (Ra), which is still a primary reference parameter used today. Roughness is one specific critical parameter that defines how much a surface measurement deviates from a specified shape or form, with height variation within the millimeter lateral range. For these reasons, for the nanocomposites containing 5% by weight of ZnO, AlZnO, TiO_2_, ZrO_2_, Al_2_O_3_, SiO_2_ and kaolin nanoclay, surface tests were performed on the fabricated prints. The results of the mean absolute deviation of roughness and waviness for individual samples (Figures S85–S91) are given in Table 6. Figure 17 shows an example of the surface topography of the nanocomposite containing 5% by weight silicon oxide, while Figure 18 shows the roughness (Figure 18a) and waviness (Figure 18b) of the tested surface of the silicon oxide print. Measurements for the composite with 5 wt.% silicon oxide were made in alignment B, according to Figure 3. 

**Figure 19:**
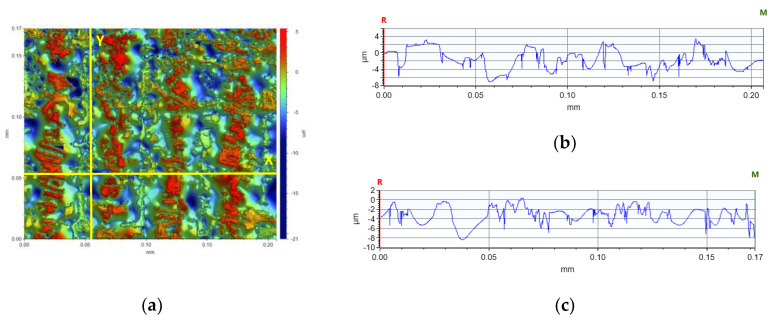
Analysis of the height of the tested surface of the print (**a**) from the base blends of monomers polymerizing according to the radical mechanism in the (**b**) *X*-axis (∆X = 0.2067 mm, ∆Z = −2.0549 µm) and in the (**c**) *Y*-axis (∆X = 0.1722 mm, ∆Z = −1.7119 µm).

In the comparison of the surface parameters of the tested samples (Table 5), one can see the influence of individual nanoadditives on the roughness and waviness of the sample. Based on the analysis, it is clear that with this type of test it is possible to determine the quality of 3D-VAT printed objects. With this analysis, we can analyze how the addition of nanoparticles affects the resolution of the 3D-VAT printing process. The results of the average absolute deviation of the surface roughness varied from 0.232 [µm] for the nanocomposition with a 5% weight content of TiO_2_ to 1.888 [µm] for the base composition. The results of the mean absolute deviation for the waviness of the tested surface oscillated between the value of 1.674 [µm] for the base composition and 0.060 [µm] obtained for the nanocomposition with 5% weight content of titanium nano-oxide. The highest values of chromaticity of the surface of the printed objects were achieved for the photocurable resin that did not contain any nanoadditives, which indicated that the photocurable resin in the 3D printing process achieved a very good printing resolution, as shown in the images that visualize individual pixels formed during 3D printing. Figure 19 shows an analysis of the surface height of the *X*- and *Y*-axis print made from the radical base composition. It shows the printed pixel shapes created during 3D printing. Figures S92–S98 illustrate the surface heights for the other samples tested. The introduction of nanoadditives to the photocured resin resulted in a decrease in printability, which may have been due to the fact that light penetration was impaired by the addition of nanoparticles. This phenomenon occurred especially in the case of resin containing titanium nano-oxide, which strongly absorbed light radiation; thus, prints for this composition with the addition of titanium oxide had the lowest roughness and print accuracy. 

#### 3.7.2. SEM Investigation of Selected 3D Prints

**Figure 20:**
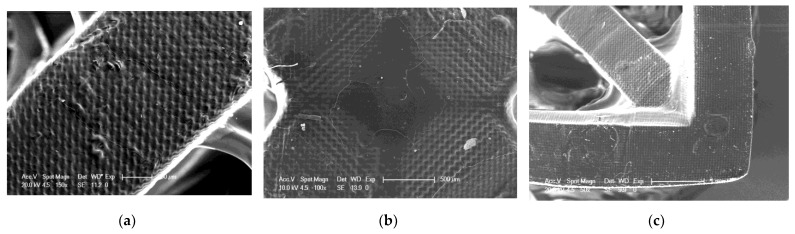
SEM of 3D printing of the base composition; (**a**) magnification 150×; (**b**) magnification 100×; magnification 50×.

**Figure 21:**
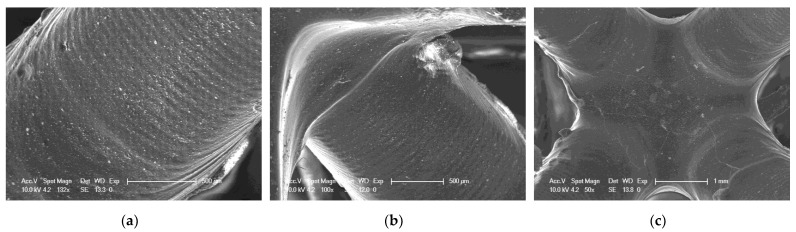
SEM of 3D printing of the nanocomposition containing 5% by weight ZrO_2_; (**a**) magnification 132×; (**b**) magnification 100×; (**c**) magnification 50×.

**Figure 22:**
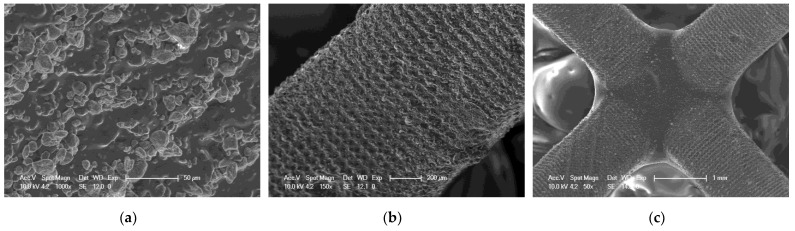
SEM of 3D printing of the nanocomposition containing 5% by weight Kaolin nanoclay; (**a**) magnification 1000×; (**b**) magnification 150×; (**c**) magnification 50×.

As part of the analysis of the quality of 3D printed objects, an analysis of the surface of the prints was performed using an SEM. SEM analysis was performed for three selected compositions. The selected compositions were as follows: a base composition consisting of a TPO-L photoinitiator and UDMA/TEGDMA monomers mixed relative to each other in a weight ratio of 7:3; a nanocomposition containing 5% by weight of ZrO_2_ popularly used in implantology [61,62]; and a nanocomposition containing 5% by weight of Kaolin nanoclay. The results obtained are summarized in Figure 20, Figure 21 and Figure 22. 

It can be seen that both the resin with the addition of kaolin nanoclay (Figure 22) and the base resin (Figure 20) achieved similar resolution at the given parameters, which was also confirmed with tests performed with a profilometer (Table 5). Figure 20, Figure 21 and Figure 22 clearly show the position of the individual print layers. Figure 22a shows the nanofiller agglomerates located in the nanocomposition. The best resolution was observed for the base composition consisting of a blend of pure monomers. For the nanofiller in the form of ZrO_2_, a deterioration in resin resolution was evident (Figure 21a–c).

## 4. Conclusions

This article has presented new polymerization compositions, using both radical and cationic mechanisms, for the preparation of dental composites, as well as for 3D printing.

It has been shown that inorganic fillers, such as ZnO, AlZnO, TiO_2_, ZrO_2_, Al_2_O_3_, SiO_2_, and kaolin nanoclay, generally led to a decrease in the rate of both cationic and radical photopolymerization, which was caused by the limited light penetration in the composition. Nevertheless, high-performance initiators were used in this study, which allowed high conversion rates of over 70% to be achieved for all compositions tested. In addition, the viscosities of the nanocomposites produced in this study were similar to those of the commercial dental resin-type products. Kinetic studies of the polymer nanocomposites showed that the use of such nanoadditives as ZnO, AlZnO, TiO_2_, ZrO_2_, and Al_2_O_3_ nano-oxides in the radical reaction system influenced the white color of the cured composites. The developed composites polymerizing according to the radical mechanism have also been proposed to obtain high-resolution 3D printouts, as well as composites dedicated to the dental industry. 

On one hand, the process of radical photopolymerization is fast and allows us to obtain composites with excellent mechanical properties. On the other hand, in this type of polymerization, there is an unfavorable phenomenon of oxygen inhibition and often undesirable polymerization shrinkage. Acrylate monomers polymerizing according to the radical mechanism also often cause allergies, as well as being toxic. Therefore, a promising alternative for the dental industry and 3D printing is the cationic photopolymerization process, where in this case nontoxic monomers are used, and the composites have low polymerization shrinkage. Nevertheless, in this case, composites with inferior mechanical properties were obtained, so further detailed studies are still required.

## Figures and Tables

**Table 1:** Absorbance characteristics of applied photoinitiators.

Acronym	$\mathrm{Extinction}\;\mathrm{at}\;\mathrm{the}\;\mathrm{Longest}\;\mathrm{Band}\;\left[dm^{3}\cdot mol^{-1}\cdot cm^{-1}\right]$	$\lambda_{max-ab}$ at the Longest Band [nm]	$\mathrm{Extinction}\;\mathrm{at}\;405\;\mathrm{nm}\;\left[dm^{3}\cdot mol^{-1}\cdot cm^{-1}\right]$
TPO-L	239	371	83
Sylanto 7 MP	17,347	349	105

**Table 2:** Summary of viscosity values for radical and cationic compositions and nanocompositions.

Composition	Radical Polymerization	Cationic Polymerization
	Viscosity [mPa·s]	
UDMA/TEGDMA 7:3 + TPO-L	286	-
VBT + Sylanto 7 MP	-	216
1% *w/w* ZnO	315	294
5% *w/w* ZnO	334	-
1% *w/w* AlZnO	311	295
5% *w/w* AlZnO	324	-
0.1% *w/w* TiO_2_	290	232
0.5% *w/w* TiO_2_	297	-
10% *w/w* TiO_2_	369	-
50% *w/w* TiO_2_	768	-
1% *w/w* Kaolin nanoclay	284	392
5% *w/w* Kaolin nanoclay	377	-
10% *w/w* Kaolin nanoclay	374	-
50% *w/w* Kaolin nanoclay	1839	-
1% *w/w* ZrO_2_	312	-
5% *w/w* ZrO_2_	335	-
10% *w/w* ZrO_2_	336	-
50% *w/w* ZrO_2_	660	-
1% *w/w* Al_2_O_3_	293	-
5% *w/w* Al_2_O_3_	346	-
10% *w/w* Al_2_O_3_	383	-
50% *w/w* Al_2_O_3_	3485	-
1% *w/w* SiO_2_	342	-
5% *w/w* SiO_2_	539	-
10% *w/w* SiO_2_	6781	-

**Table 3:** Summary of results for measurements of kinetics and conversion rates of photopolymerization of radical-reactive nanocomposites.

Composition	1.4 mm			25 μm		
	Conversion [%]	Induction Time [s]	$\mathrm{Slope}\;\mathrm{of}\;\mathrm{the}\;\mathrm{Kinetic}\;\mathrm{Curve}\;[s^{-1}]$	Conversion [%]	Induction Time [s]	$\mathrm{Slope}\;\mathrm{of}\;\mathrm{the}\;\mathrm{Kinetic}\;\mathrm{Curve}\;[s^{-1}]$
UDMA/TEGDMA 7:3 + TPO-L	86	14	6.66	73	11	4.74
1% *w/w* ZnO	80	11	3.93	76	12	7.16
5% *w/w* ZnO	82	17	1.74	76	13	9.24
1% *w/w* AlZnO	81	11	3.83	77	12	6.31
5% *w/w* AlZnO	79	11	3.73	79	12	6.50
0.1% *w/w* TiO_2_	85	12	5.10	75	13	6.53
0.5% *w/w* TiO_2_	78	17	4.24	77	12	7.99
1% *w/w* Kaolin nanoclay	84	14	6.66	70	13	4.82
5% *w/w* Kaolin nanoclay	84	14	6.63	76	15	6.93
1% *w/w* ZrO_2_	72	17	2.67	71	17	4.50
5% *w/w* ZrO_2_	71	20	3.07	74	15	4.02
1% *w/w* Al_2_O_3_	77	19	3.49	70	21	2.66
5% *w/w* Al_2_O_3_	75	23	6.00	71	21	3.56
1% *w/w* SiO_2_	70	19	3.55	71	19	2.93
5% *w/w* SiO_2_	74	21	3.93	70	19	2.61

**Table 4:** Summary of results for measurements of kinetics and conversion rates of photopolymerization of cationic-reactive nanocomposites.

Composition	1.4 mm			25 μm		
	Conversion [%]	Induction Time [s]	$\mathrm{Slope}\;\mathrm{of}\;\mathrm{the}\;\mathrm{Kinetic}\;\mathrm{Curve}\;[s^{-1}]$	Conversion [%]	Induction Time [s]	$\mathrm{Slope}\;\mathrm{of}\;\mathrm{the}\;\mathrm{Kinetic}\;\mathrm{Curve}\;[s^{-1}]$
VBT + Sylanto 7 MP	83	69	0.67	83	108	0.80
1% *w/w* ZnO	78	94	-	80	231	0.42
1% *w/w* AlZnO	84	30	1.15	84	188	0.63
0.1% *w/w* TiO_2_	78	79	0.61	80	145	1.05
1% *w/w* Kaolin nanoclay	83	158	1.18	80	197	0.85

**Table 5:** Summary of nanocomposite color before and after being cured under a dental lamp.

Before Curing	After Curing	Before Curing	After Curing
Base radical composition		10% *w/w* SiO_2_	
-	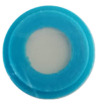	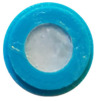	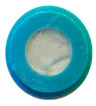
10% *w/w* TiO_2_		50% *w/w* TiO_2_	
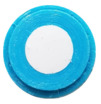	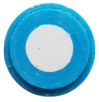	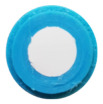	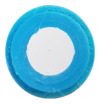
10% *w/w* Kaolin nanoclay		50% *w/w* Kaolin nanoclay	
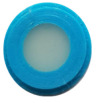	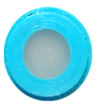	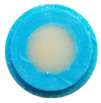	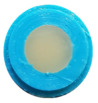
10% *w/w* ZrO_2_		50% *w/w* ZrO_2_	
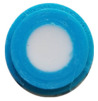	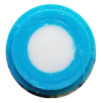	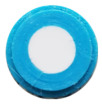	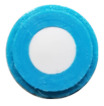
10% *w/w* Al_2_O_3_		50% *w/w* Al_2_O_3_	
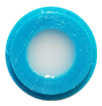	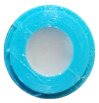	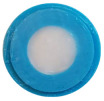	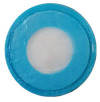

**Table 6:** Overview of the results of the mean absolute deviation for waviness and roughness of the printed surface for different nanocomposites.

**Composition**	**Waviness [µm]**	**Roughness [µm]**
Base	1.674	1.888
5% *w/w* ZnO	0.161	0.329
5% *w/w* AlZnO	0.472	0.497
5% *w/w* TiO_2_	0.060	0.232
5% *w/w* Kaolin nanoclay	0.639	1.301
5% *w/w* ZrO_2_	0.169	0.347
5% *w/w* Al_2_O_3_	0.394	1.125
5% *w/w* SiO_2_	0.434	0.860

**Composition**

**Waviness [µm]**

**Roughness [µm]**

## Data Availability

Noworyta, Małgorzata; Topa-Skwarczyńska, Monika; Jamróz, Paweł; Oksiuta, Dawid; Tyszka-Czochara, Małgorzata; Trembecka-Wójciga, Klaudia; Ortyl, Joanna (2023), “Influence of the Type of Nanofillers on the Properties of Composites Used in Dentistry and 3D Printing”, Mendeley Data, V1, https://doi.org/17632/x9frkd5ync.1.

## Supplementary Materials

The supporting information can be downloaded at: https://www.mdpi.com/article/10.3390/ijms241310549/s1.
