# Supplementary material for: Influence of the Type of Nanofillers on the Properties of Composites Used in Dentistry and 3D Printing
Source: Int J Mol Sci. 2023 Jun 23;24(13):10549. doi: 10.3390/ijms241310549 (PMC10341704; doi:10.3390/ijms241310549)
Supplement: Supplementary file 1 [file ijms-24-10549-s001.zip › ijms-2415901-supplementary.pdf]

Supplementary Materials

Influence of the type of nanofillers on the properties of composites used in dentistry and 3D printing

Małgorzata Noworyta [1], Monika Topa-Skwarczyńska [1], Paweł Jamróz [1], Dawid Oksiuta [2], Małgorzata Tyszka-Czochara [3], Klaudia Trembecka -Wójciga [4], Joanna Ortyl [1,5,6*]

[1] Cracow University of Technology, Faculty of Chemical Engineering and Technology, Warszawska 24, 31- 155 Cracow, Poland

[2] Cracow University of Technology, Faculty of Mechanical Engineering, Jana Pawła II 37, 31-864 Cracow, Poland

[3] Faculty of Pharmacy, Jagiellonian University Medical College, Medyczna 9, 30-688 Kraków, Poland

[4] Institute of Metallurgy and Materials Science, Polish Academy of Sciences, Reymonta 25, 30-059 Cracow, Poland

[5] Photo4Chem Ltd., Lea 114, 31-133 Cracow, Poland

[6] Photo HiTech Ltd., Bobrzyńskiego 14, 30-348 Cracow, Poland

[*] Correspondence: jortyl@chemia.pk.edu.pl

The dependence of the viscosity of radical-reactive compositions on the shear rate for a constant temperature of 25°C.

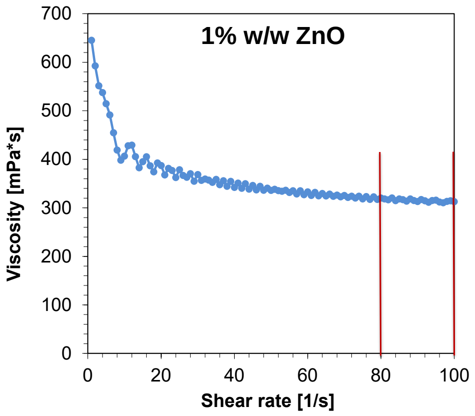

Figure S1. Dependence of viscosity, nanocomposition polymerizing by radical mechanism with 1% wt. ZnO on shear rate for a constant temperature of 25°C.

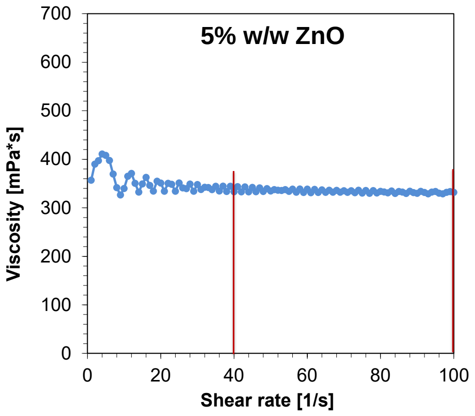

Figure S2. Dependence of viscosity, nanocomposition polymerizing by radical mechanism with 5% wt. ZnO on shear rate for a constant temperature of 25°C.

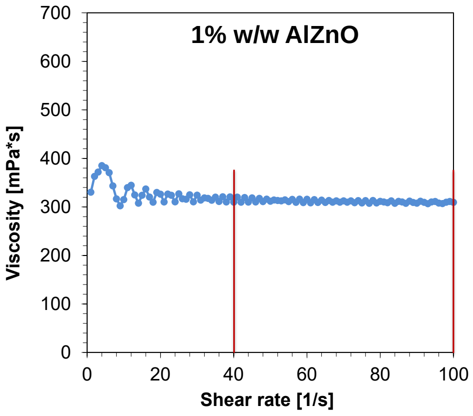

Figure S3. Dependence of viscosity, nanocomposition polymerizing by radical mechanism with 1% wt. AlZnO on shear rate for a constant temperature of 25°C.

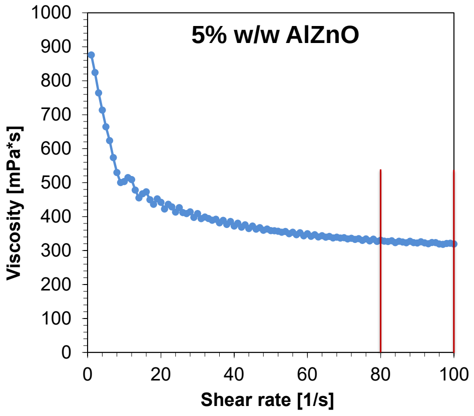

Figure S4. Dependence of viscosity, nanocomposition polymerizing by radical mechanism with 5% wt. AlZnO on shear rate for a constant temperature of 25°C.

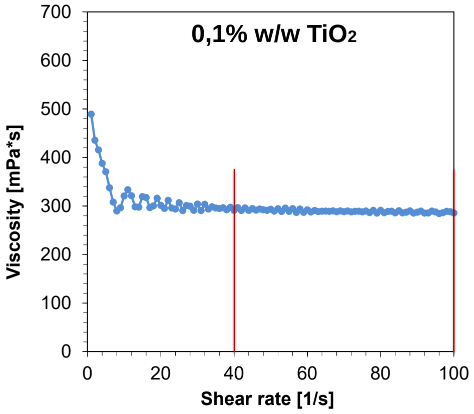

Figure S5. Dependence of viscosity, nanocomposition polymerizing by radical mechanism with 0,1% wt. TiO_2 on shear rate for a constant temperature of 25°C.

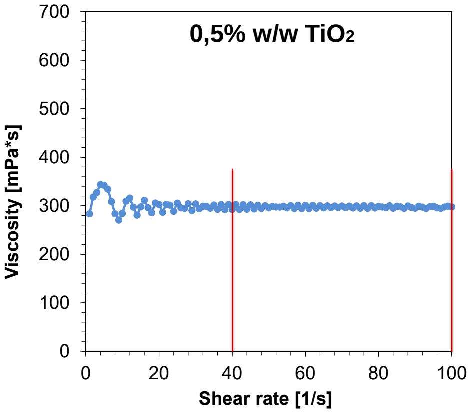

Figure S6. Dependence of viscosity, nanocomposition polymerizing by radical mechanism with 0,5% wt. TiO_2 on shear rate for a constant temperature of 25°C.

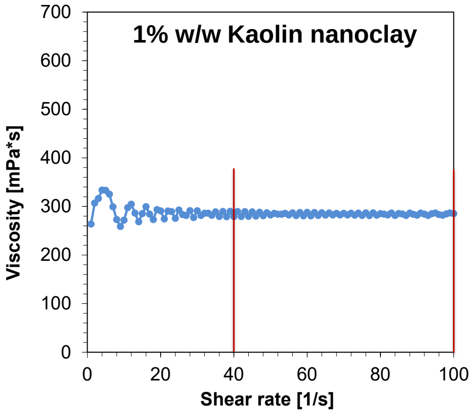

Figure S7. Dependence of viscosity, nanocomposition polymerizing by radical mechanism with 1% wt. Kaolin nanoclay on shear rate for a constant temperature of 25°C.

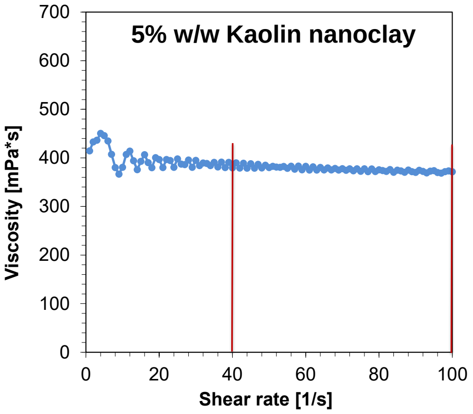

Figure S8. Dependence of viscosity, nanocomposition polymerizing by radical mechanism with 5% wt. Kaolin nanoclay on shear rate for a constant temperature of 25°C.

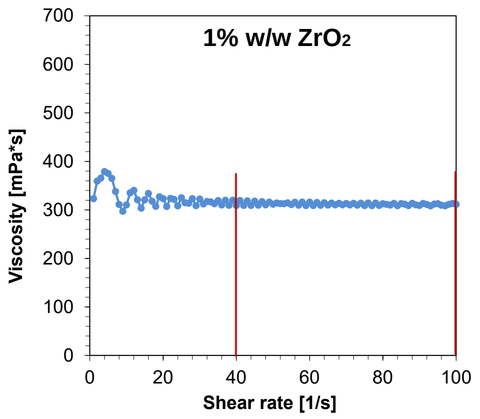

Figure S9. Dependence of viscosity, nanocomposition polymerizing by radical mechanism with 1% wt. ZrO_2 on shear rate for a constant temperature of 25°C.

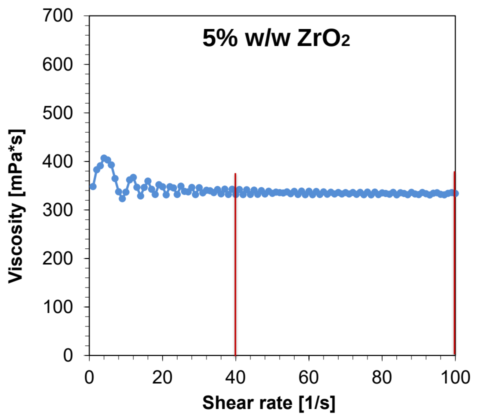

Figure S10. Dependence of viscosity, nanocomposition polymerizing by radical mechanism with 5% wt. ZrO_2 on shear rate for a constant temperature of 25°C.

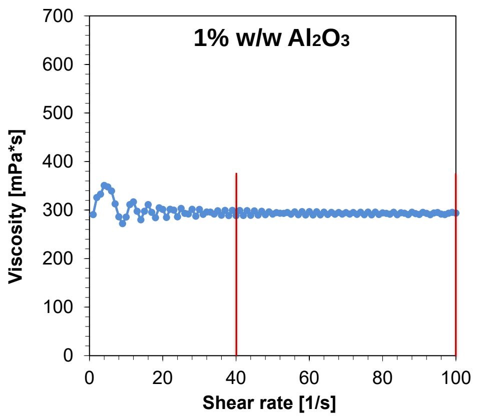

Figure S11. Dependence of viscosity, nanocomposition polymerizing by radical mechanism with 1% wt. Al_2O_3 on shear rate for a constant temperature of 25°C.

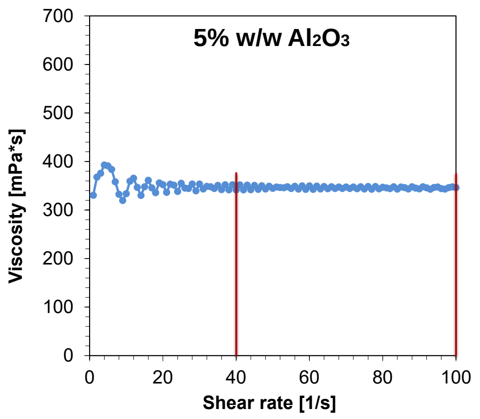

Figure S12. Dependence of viscosity, nanocomposition polymerizing by radical mechanism with 5% wt. Al_2O_3 on shear rate for a constant temperature of 25°C.

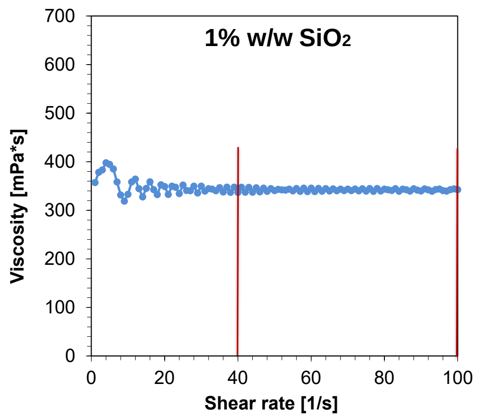

Figure S13. Dependence of viscosity, nanocomposition polymerizing by radical mechanism with 1% wt. SiO_2 on shear rate for a constant temperature of 25°C.

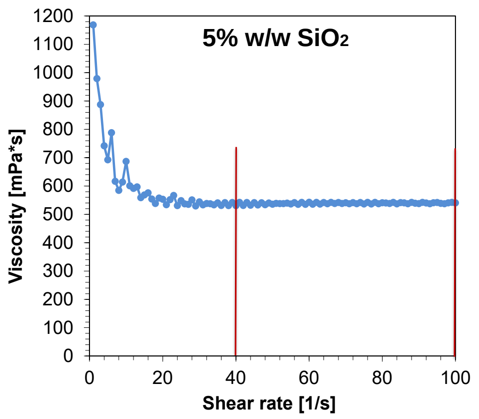

Figure S14. Dependence of viscosity, nanocomposition polymerizing by radical mechanism with 5% wt. SiO_2 on shear rate for a constant temperature of 25°C.

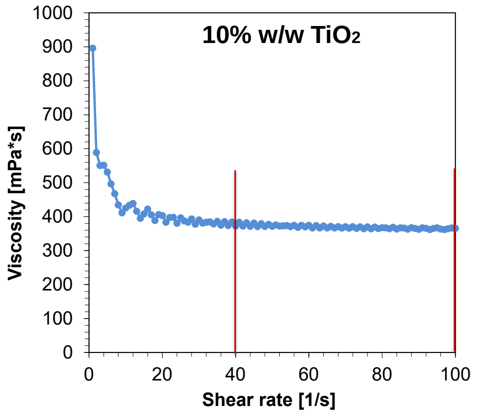

Figure S15. Dependence of viscosity, nanocomposition polymerizing by radical mechanism with 10% wt. TiO_2 on shear rate for a constant temperature of 25°C.

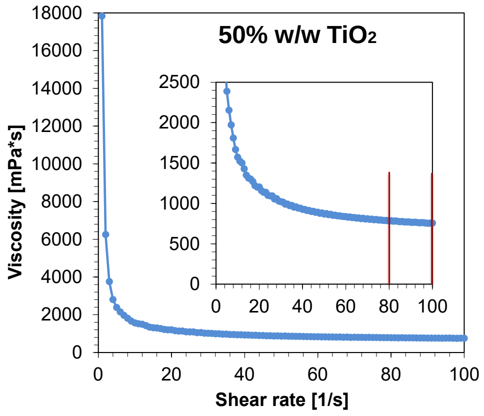

Figure S16. Dependence of viscosity, nanocomposition polymerizing by radical mechanism with 50% wt. TiO_2 on shear rate for a constant temperature of 25°C.

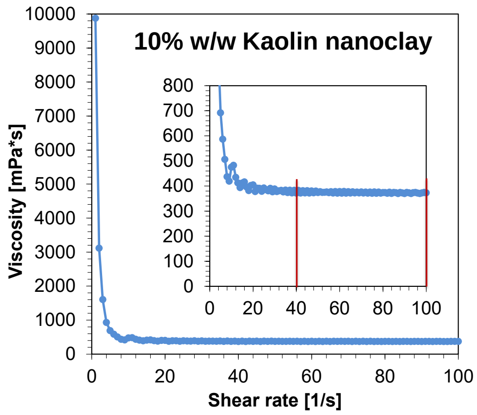

Figure S17. Dependence of viscosity, nanocomposition polymerizing by radical mechanism with 10% wt. Kaolin nanoclay on shear rate for a constant temperature of 25°C.

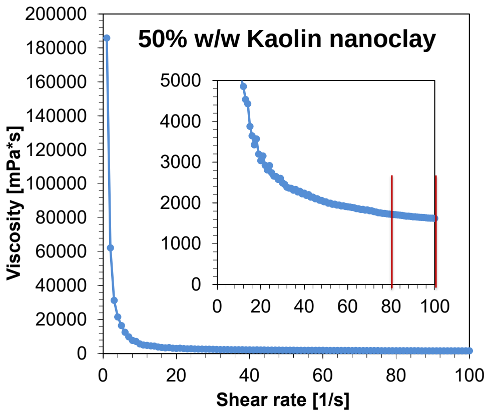

Figure S18. Dependence of viscosity, nanocomposition polymerizing by radical mechanism with 50% wt. Kaolin nanoclay on shear rate for a constant temperature of 25°C.

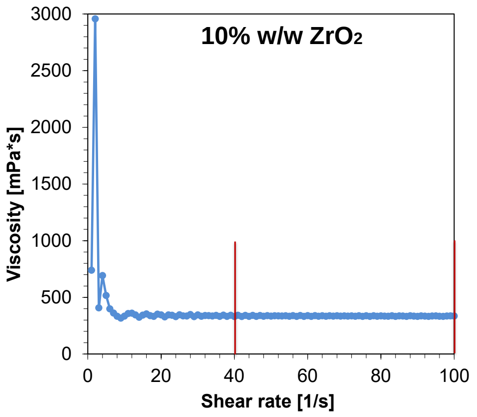

Figure S19. Dependence of viscosity, nanocomposition polymerizing by radical mechanism with 10% wt. ZrO_2 on shear rate for a constant temperature of 25°C.

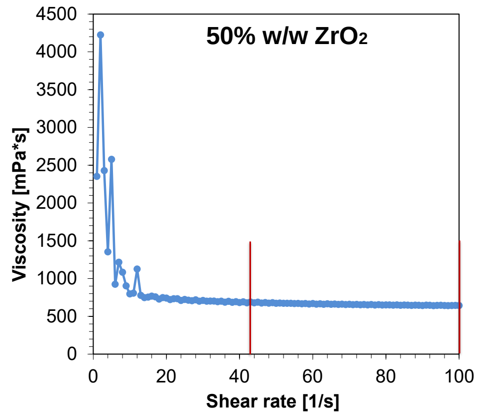

Figure S20. Dependence of viscosity, nanocomposition polymerizing by radical mechanism with 50% wt. ZrO_2 on shear rate for a constant temperature of 25°C.

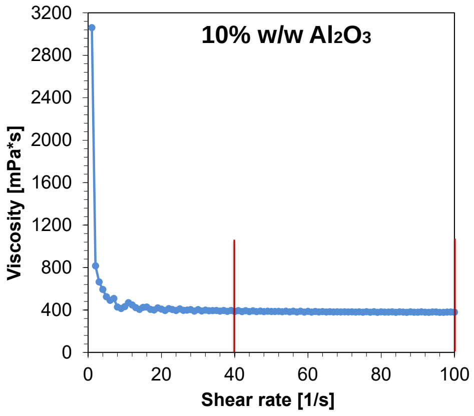

Figure S21. Dependence of viscosity, nanocomposition polymerizing by radical mechanism with 10% wt. Al_2O_3 on shear rate for a constant temperature of 25°C.

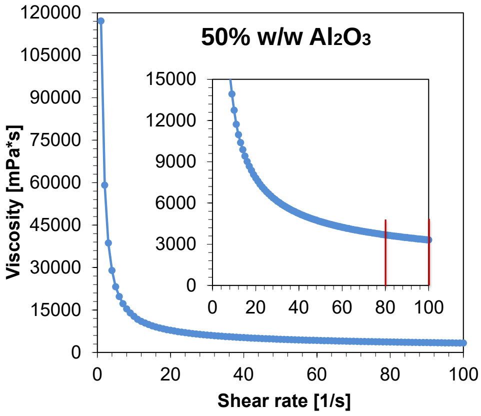

Figure S22. Dependence of viscosity, nanocomposition polymerizing by radical mechanism with 50% wt. Al_2O_3 on shear rate for a constant temperature of 25°C.

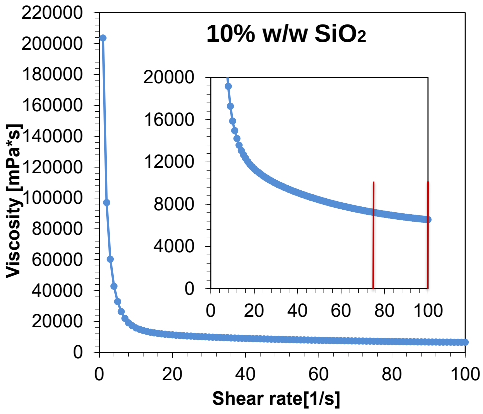

Figure S23. Dependence of viscosity, nanocomposition polymerizing by radical mechanism with 10% wt. SiO_2 on shear rate for a constant temperature of 25°C.

The dependence of the viscosity of cationic-reactive compositions on the shear rate for a constant temperature of 25°C.

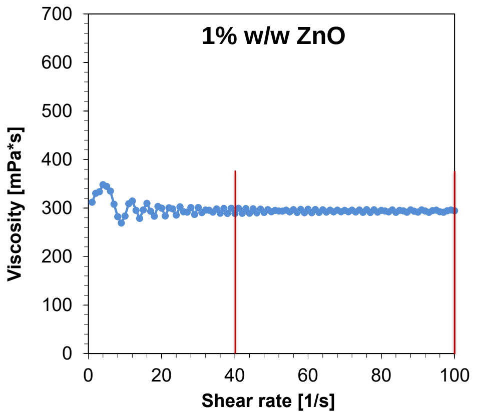

Figure S24. Dependence of viscosity, nanocomposition polymerizing by cationic mechanism with 1% wt. ZnO on shear rate for a constant temperature of 25°C.

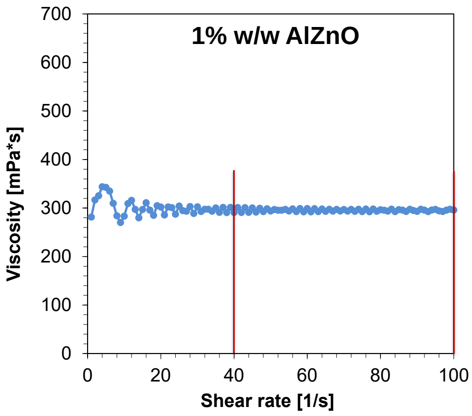

Figure S25. Dependence of viscosity, nanocomposition polymerizing by cationic mechanism with 1% wt. AlZnO on shear rate for a constant temperature of 25°C.

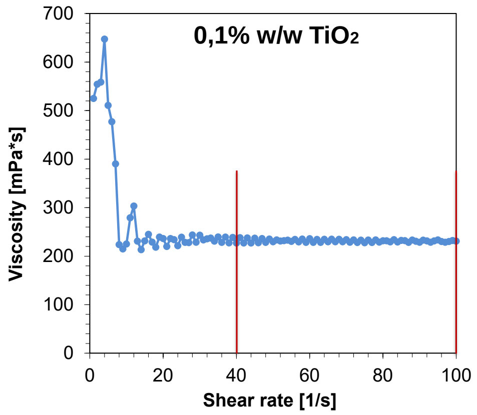

Figure S26. Dependence of viscosity, nanocomposition polymerizing by cationic mechanism with 0,1% wt. TiO_2 on shear rate for a constant temperature of 25°C.

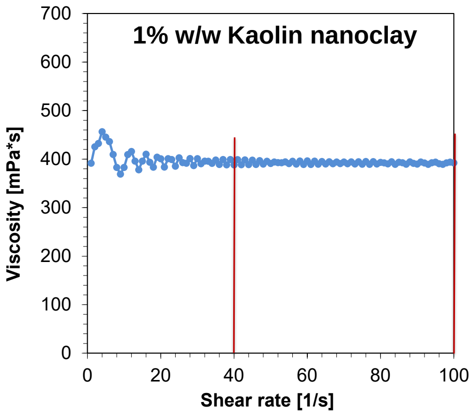

Figure S27. Dependence of viscosity, nanocomposition polymerizing by cationic mechanism with 1% wt. Kaolin nanoclay on shear rate for a constant temperature of 25°C.

Conversion rates of the photopolymerization process carried out for layers of 25 μm radical-reactive compositions containing different weight concentrations of nano-additives.

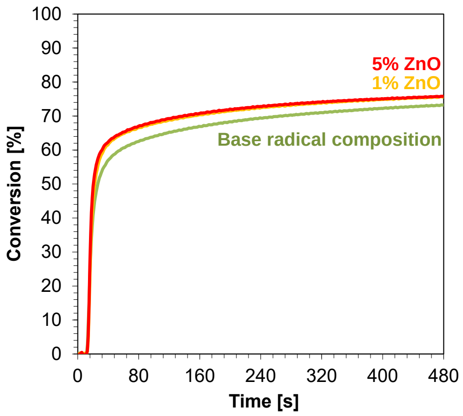

Figure S28. Kinetic profiles for compositions consisting of 1% wt. and 5% wt. ZnO for a radical reacting system and a layer thickness of 25 μm.

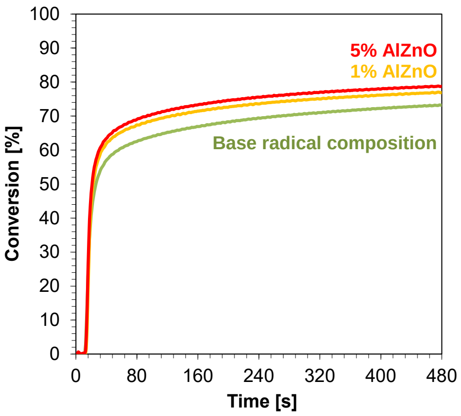

Figure S29. Kinetic profiles for compositions consisting of 1% wt. and 5% wt. AlZnO for a radical reacting system and a layer thickness of 25 μm.

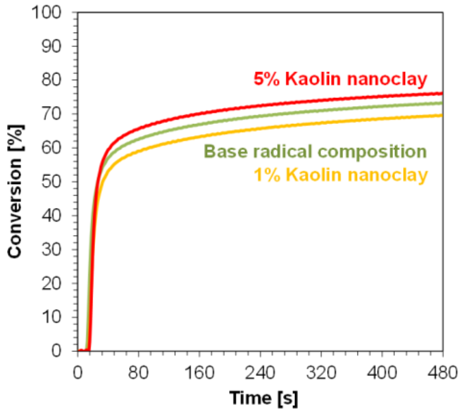

Figure S30. Kinetic profiles for compositions consisting of 1% wt. and 5% wt. Kaolin nanoclay for a radical reacting system and a layer thickness of 25 μm.

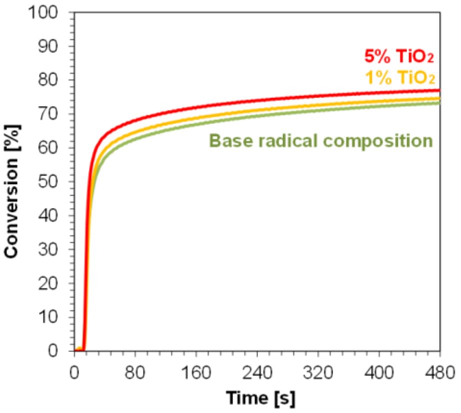

Figure S31. Kinetic profiles for compositions consisting of 1% wt. and 5% wt. TiO2 for a radical reacting system and a layer thickness of 25 μm.

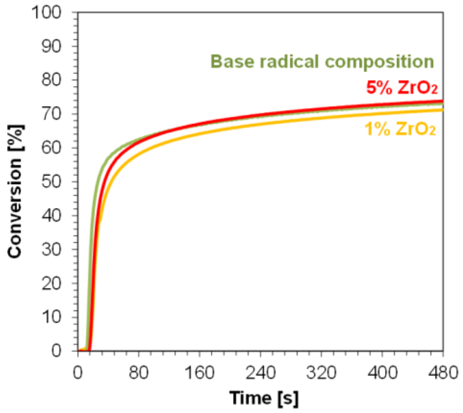

Figure S32. Kinetic profiles for compositions consisting of 1% wt. and 5% wt. ZrO2 for a radical reacting system and a layer thickness of 25 μm.

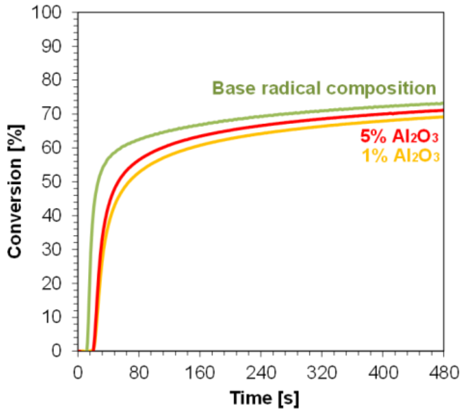

Figure S33. Kinetic profiles for compositions consisting of 1% wt. and 5% wt. Al_2O_3 for a radical reacting system and a layer thickness of 25 μm.

Conversion rates of the photopolymerization process carried out for layers of 1,4 mm radical-reactive compositions containing different weight concentrations of nano-additives.

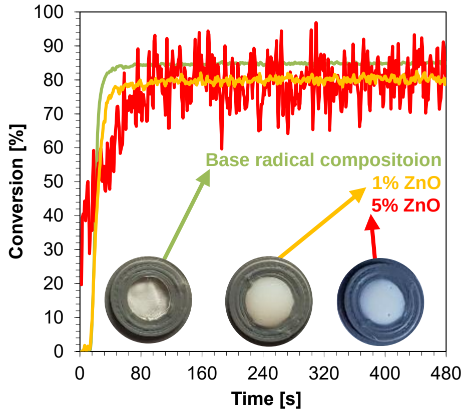

Figure S34. Kinetic profiles for compositions consisting of 1% wt. and 5% wt. ZnO for a radical reacting system and a layer thickness of 1,4 mm.

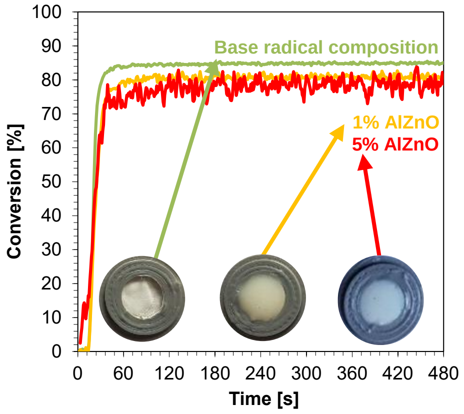

Figure S35. Kinetic profiles for compositions consisting of 1% wt. and 5% wt. AlZnO for a radical reacting system and a layer thickness of 1,4 mm.

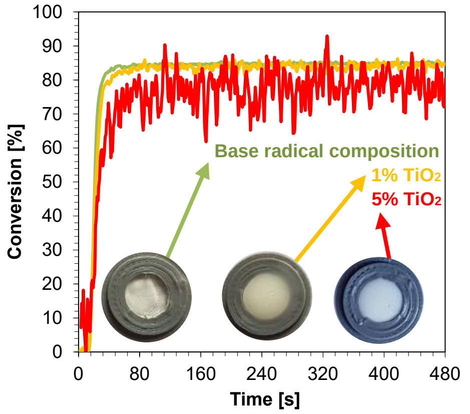

Figure S36. Kinetic profiles for compositions consisting of 0,1% wt. and 0,5% wt. TiO_2 for a radical reacting system and a layer thickness of 1,4 mm.

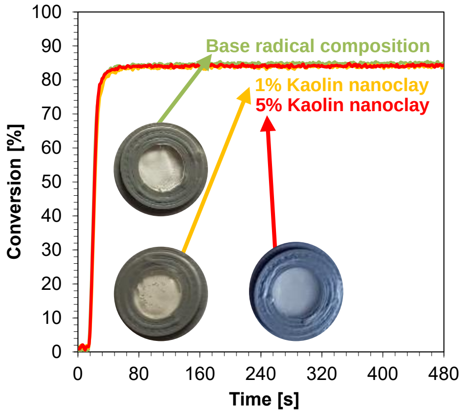

Figure S37. Kinetic profiles for compositions consisting of 1% wt. and 5% wt. Kaolin nanoclay for a radical reacting system and a layer thickness of 1,4 mm.

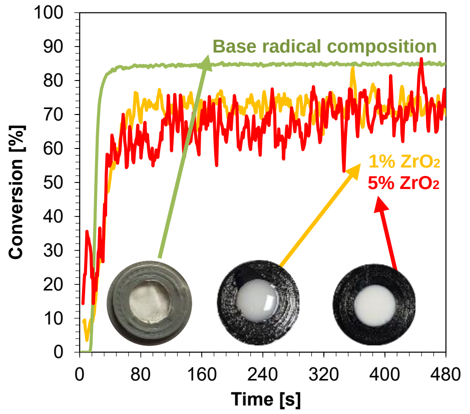

Figure S38. Kinetic profiles for compositions consisting of 1% wt. and 5% wt. ZrO_2 for a radical reacting system and a layer thickness of 1,4 mm.

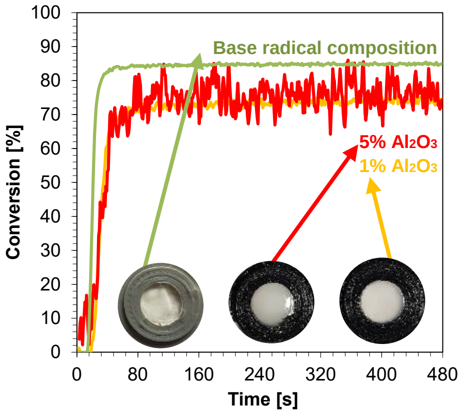

Figure S39. Kinetic profiles for compositions consisting of 1% wt. and 5% wt. Al_2O_3 for a radical reacting system and a layer thickness of 1,4 mm.

Changes in FT-IR spectra showing band decay at 1634 cm^{-1} wavenumber monitored for a radical polymerizing composition for a test layer thickness of 25 μm.

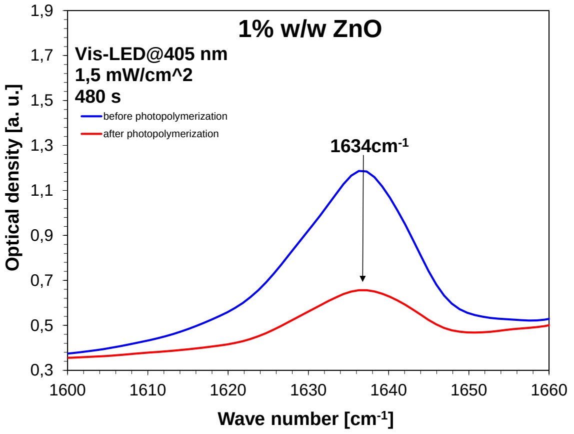

Figure S40. FT-IR spectra recorded before and after photopolymerization of nanocomposition polymerizing according to radical mechanism with 1% wt. ZnO for a 25 μm thick layer of the sample.

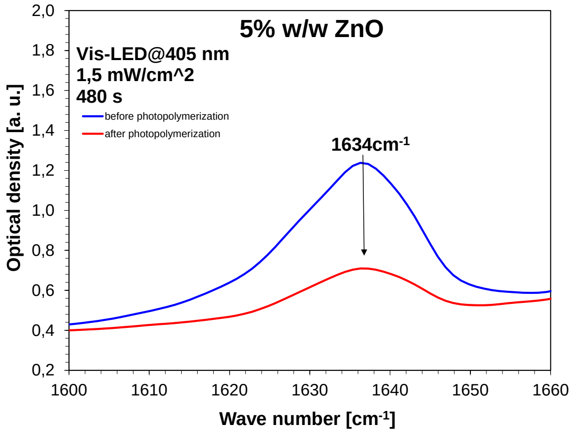

Figure S41. FT-IR spectra recorded before and after photopolymerization of nanocomposition polymerizing according to radical mechanism with 5% wt. ZnO for a 25 μm thick layer of the sample.

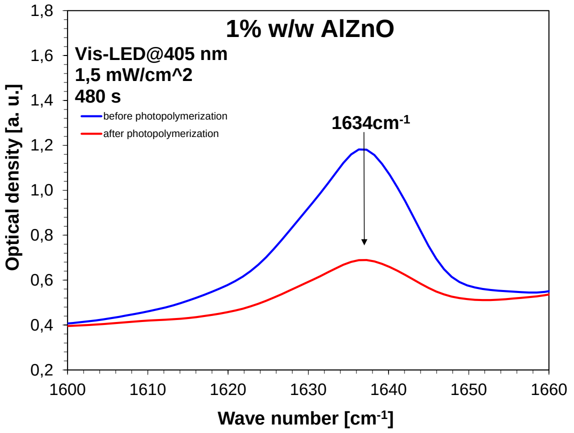

Figure S42. FT-IR spectra recorded before and after photopolymerization of nanocomposition polymerizing according to radical mechanism with 1% wt. AlZnO for a 25 μm thick layer of the sample.

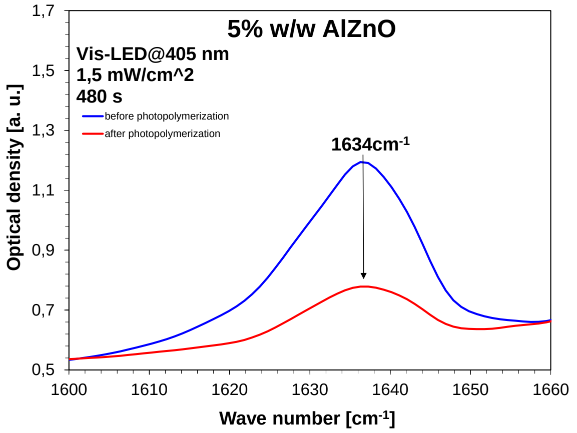

Figure S43. FT-IR spectra recorded before and after photopolymerization of nanocomposition polymerizing according to radical mechanism with 5% wt. AlZnO for a 25 μm thick layer of the sample.

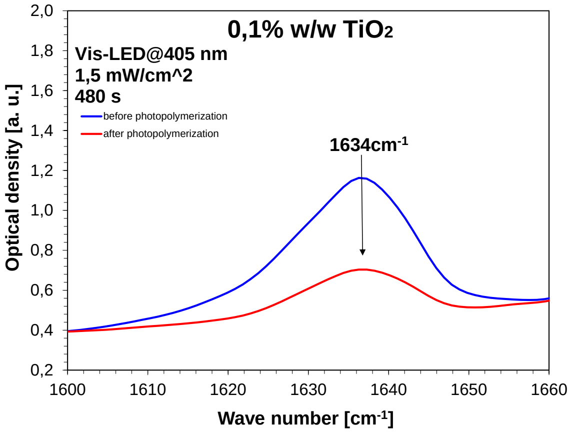

Figure S44. FT-IR spectra recorded before and after photopolymerization of nanocomposition polymerizing according to radical mechanism with 0,1% wt. TiO_2 for a 25 μm thick layer of the sample.

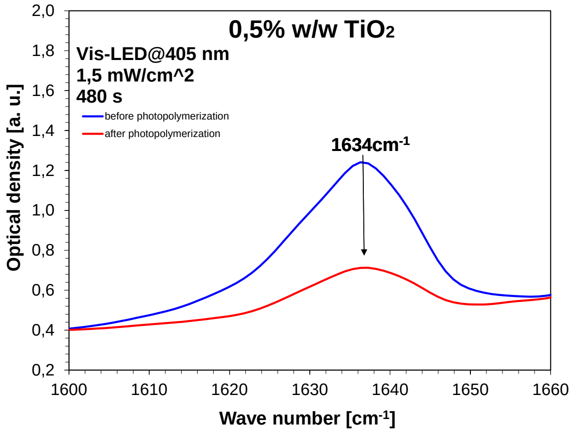

Figure S45. FT-IR spectra recorded before and after photopolymerization of nanocomposition polymerizing according to radical mechanism with 0,5% wt. TiO_2 for a 25 μm thick layer of the sample.

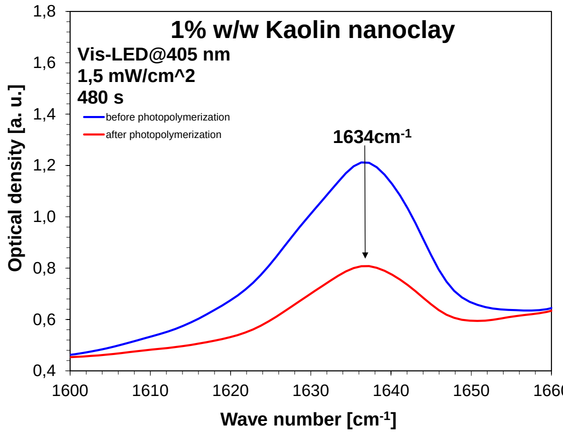

Figure S46. FT-IR spectra recorded before and after photopolymerization of nanocomposition polymerizing according to radical mechanism with 1% wt. Kaolin nanoclay for a 25 μm thick layer of the sample.

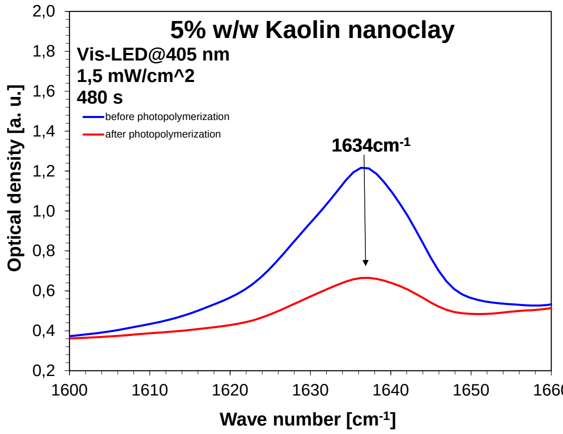

Figure S47. FT-IR spectra recorded before and after photopolymerization of nanocomposition polymerizing according to radical mechanism with 5% wt. Kaolin nanoclay for a 25 μm thick layer of the sample.

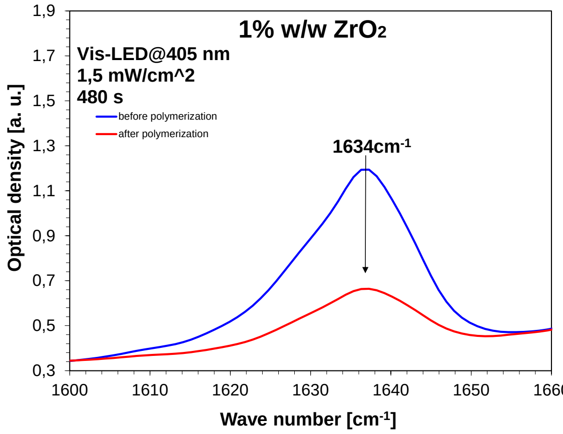

Figure S48. FT-IR spectra recorded before and after photopolymerization of nanocomposition polymerizing according to radical mechanism with 1% wt. ZrO_2 for a 25 μm thick layer of the sample.

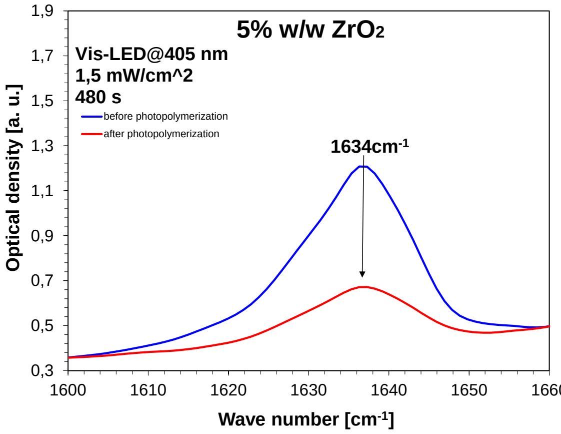

Figure S49. FT-IR spectra recorded before and after photopolymerization of nanocomposition polymerizing according to radical mechanism with 5% wt. ZrO_2 for a 25 μm thick layer of the sample.

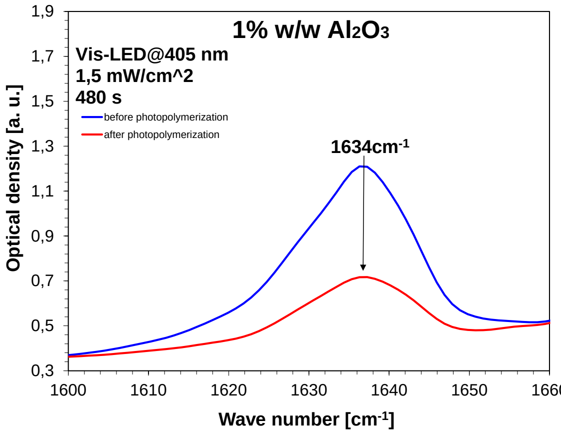

Figure S50. FT-IR spectra recorded before and after photopolymerization of nanocomposition polymerizing according to radical mechanism with 1% wt. Al_2O_3 for a 25 μm thick layer of the sample.

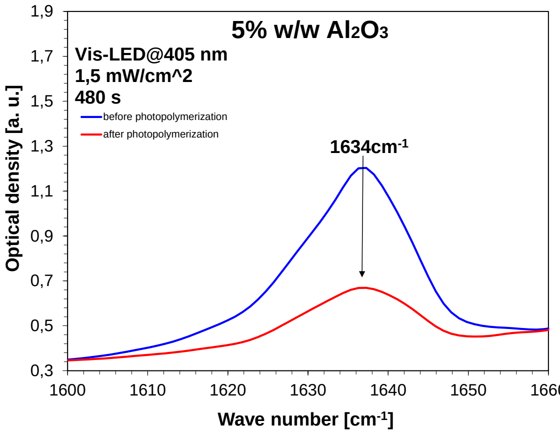

Figure S51. FT-IR spectra recorded before and after photopolymerization of nanocomposition polymerizing according to radical mechanism with 5% wt. Al_2O_3 for a 25 μm thick layer of the sample.

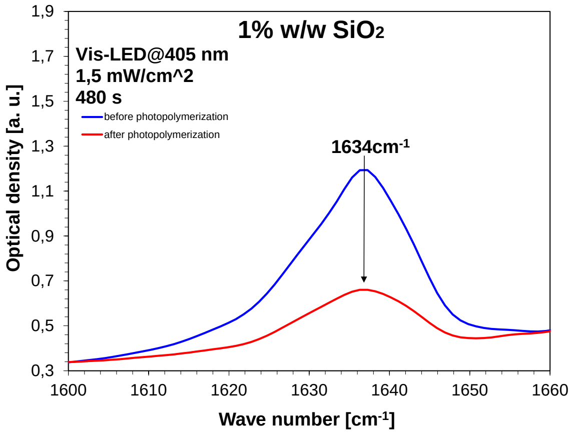

Figure S52. FT-IR spectra recorded before and after photopolymerization of nanocomposition polymerizing according to radical mechanism with 1% wt. SiO_2 for a 25 μm thick layer of the sample.

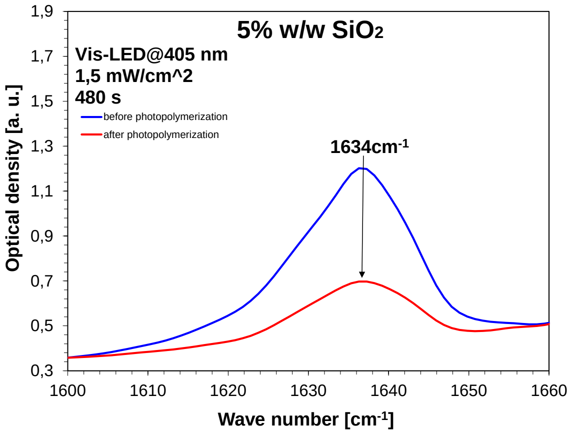

Figure S53. FT-IR spectra recorded before and after photopolymerization of nanocomposition polymerizing according to radical mechanism with 5% wt. SiO_2 for a 25 μm thick layer of the sample.

Changes in FT-IR spectra showing band decay at 1620 cm^{-1} wavenumber monitored for a cationic polymerizing composition for a test layer thickness of 25 μm.

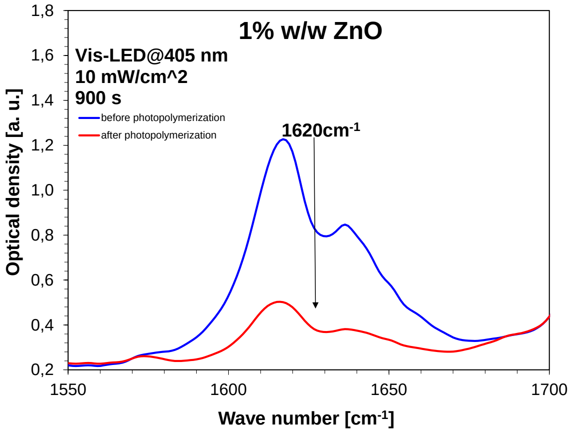

Figure S54. FT-IR spectra recorded before and after photopolymerization of nanocomposition polymerizing according to cationic mechanism with 1% wt. ZnO for a 25 μm thick layer of the sample.

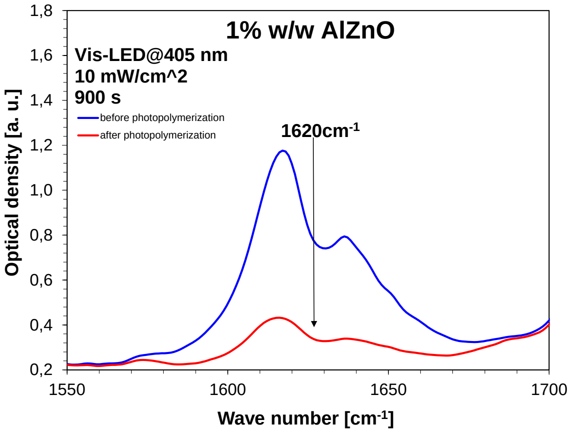

Figure S55. FT-IR spectra recorded before and after photopolymerization of nanocomposition polymerizing according to cationic mechanism with 1% wt. AlZnO for a 25 μm thick layer of the sample.

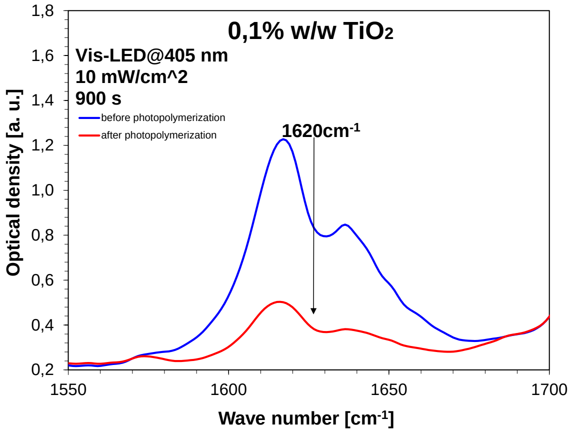

Figure S56. FT-IR spectra recorded before and after photopolymerization of nanocomposition polymerizing according to cationic mechanism with 0,1% wt. TiO_2 for a 25 μm thick layer of the sample.

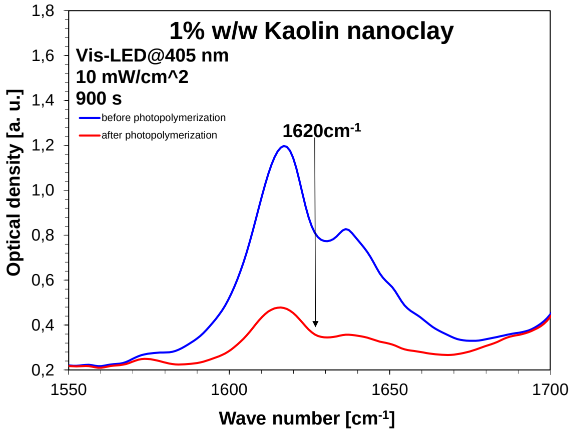

Figure S57. FT-IR spectra recorded before and after photopolymerization of nanocomposition polymerizing according to cationic mechanism with 1% wt. Kaolin nanoclay for a 25 μm thick layer of the sample.

Changes in FT-IR spectra showing band decay at 6165 cm^{-1} wavenumber monitored for a radical polymerizing composition for a test layer thickness of 1,4 mm.

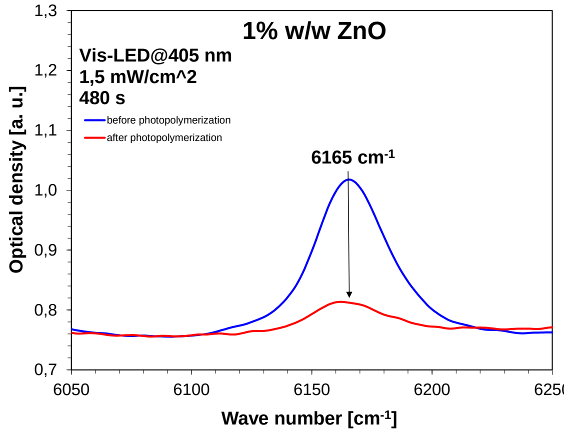

Figure S58. FT-IR spectra recorded before and after photopolymerization of nanocomposition polymerizing according to radical mechanism with 1% wt. ZnO for a 1,4 mm thick layer of the sample.

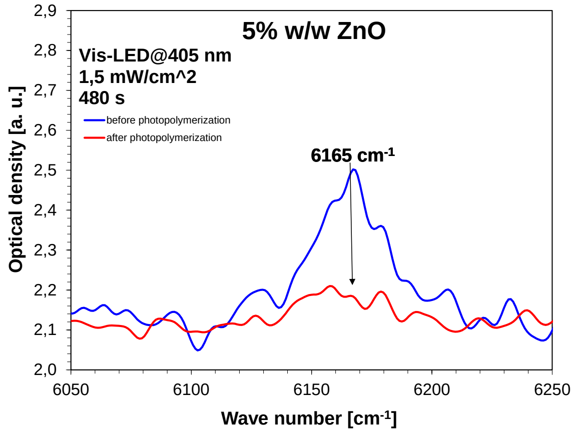

Figure S59. FT-IR spectra recorded before and after photopolymerization of nanocomposition polymerizing according to radical mechanism with 5% wt. ZnO for a 1,4 mm thick layer of the sample.

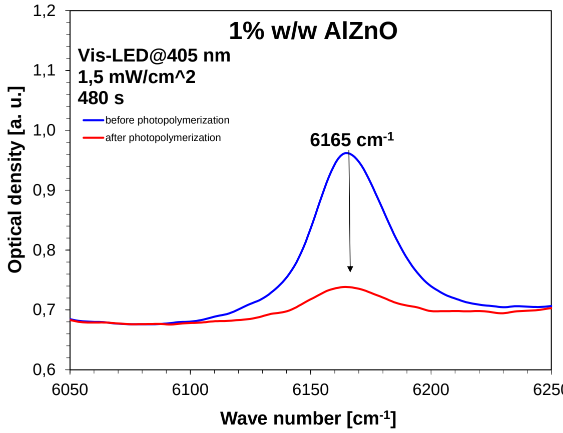

Figure S60. FT-IR spectra recorded before and after photopolymerization of nanocomposition polymerizing according to radical mechanism with 1% wt. AlZnO for a 1,4 mm thick layer of the sample.

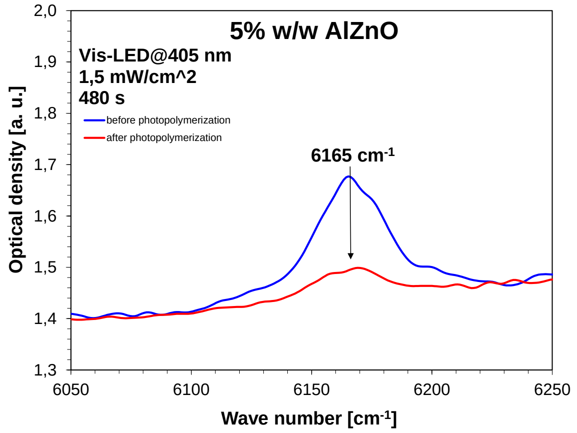

Figure S61. FT-IR spectra recorded before and after photopolymerization of nanocomposition polymerizing according to radical mechanism with 5% wt. AlZnO for a 1,4 mm thick layer of the sample.

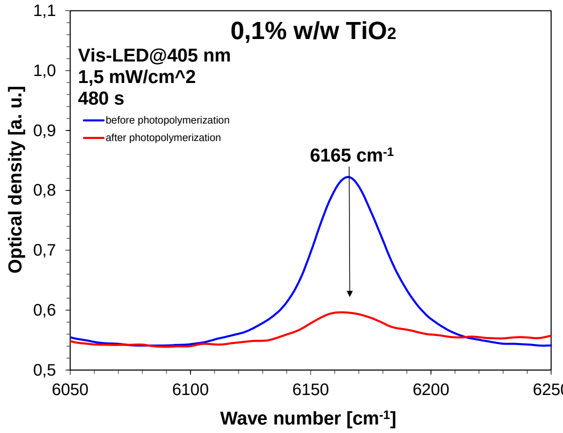

Figure S62. FT-IR spectra recorded before and after photopolymerization of nanocomposition polymerizing according to radical mechanism with 0,1% wt. TiO_2 for a 1,4 mm thick layer of the sample.

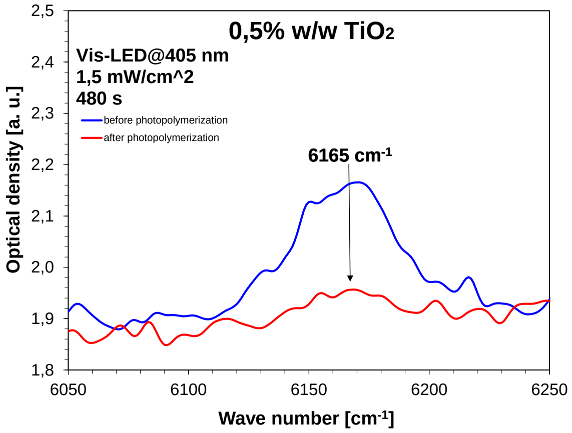

Figure S63. FT-IR spectra recorded before and after photopolymerization of nanocomposition polymerizing according to radical mechanism with 0,5% wt. TiO_2 for a 1,4 mm thick layer of the sample.

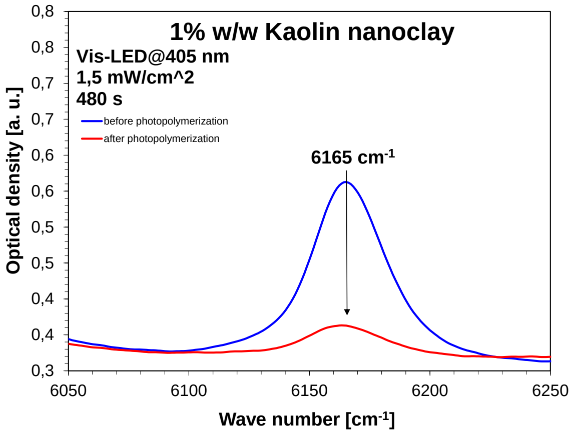

Figure S64. FT-IR spectra recorded before and after photopolymerization of nanocomposition polymerizing according to radical mechanism with 1% wt. Kaolin nanoclay for a 1,4 mm thick layer of the sample.

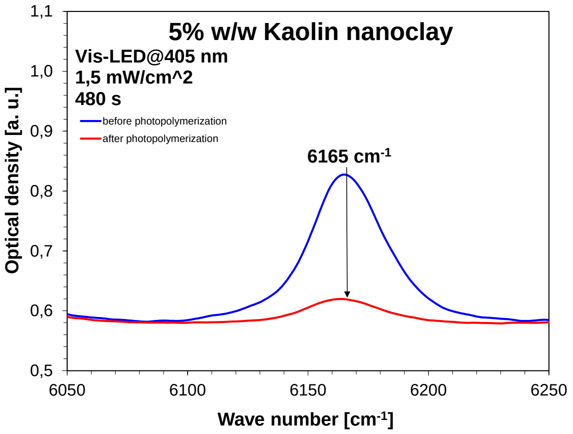

Figure S65. FT-IR spectra recorded before and after photopolymerization of nanocomposition polymerizing according to radical mechanism with 5% wt. Kaolin nanoclay for a 1,4 mm thick layer of the sample.

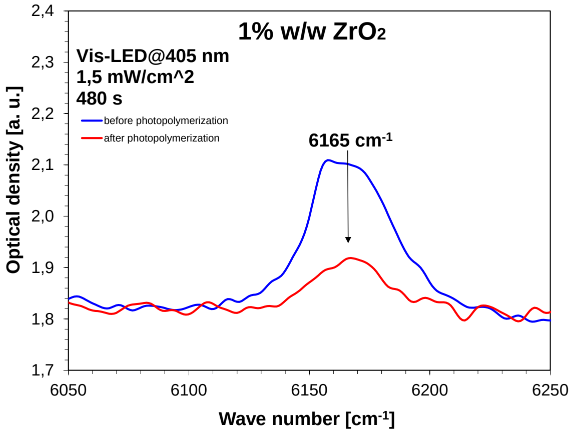

Figure S66. FT-IR spectra recorded before and after photopolymerization of nanocomposition polymerizing according to radical mechanism with 1% wt. ZrO_2 for a 1,4 mm thick layer of the sample.

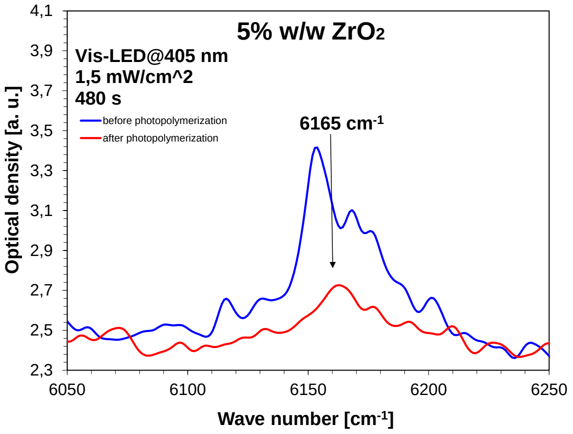

Figure S67. FT-IR spectra recorded before and after photopolymerization of nanocomposition polymerizing according to radical mechanism with 5% wt. ZrO_2 for a 1,4 mm thick layer of the sample.

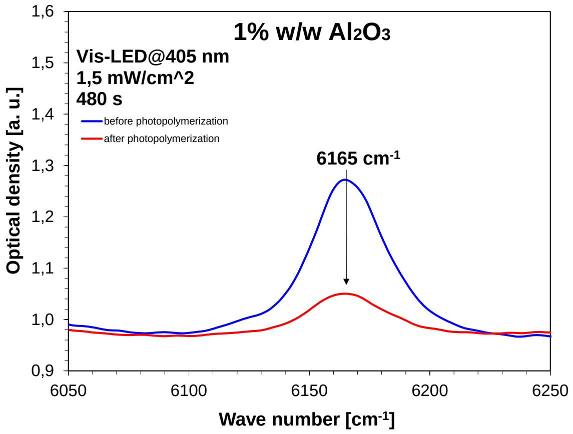

Figure S68. FT-IR spectra recorded before and after photopolymerization of nanocomposition polymerizing according to radical mechanism with 1% wt. Al_2O_3 for a 1,4 mm thick layer of the sample.

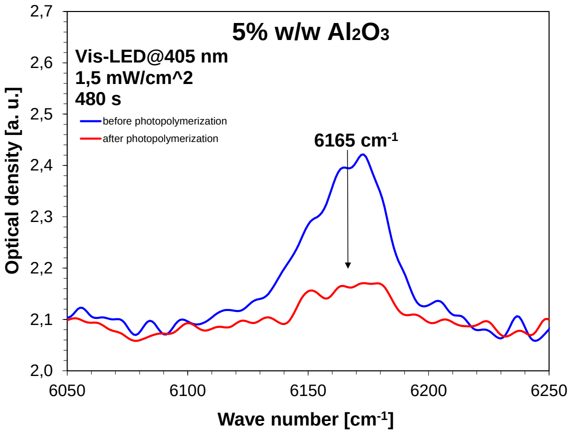

Figure S69. FT-IR spectra recorded before and after photopolymerization of nanocomposition polymerizing according to radical mechanism with 5% wt. Al_2O_3 for a 1,4 mm thick layer of the sample.

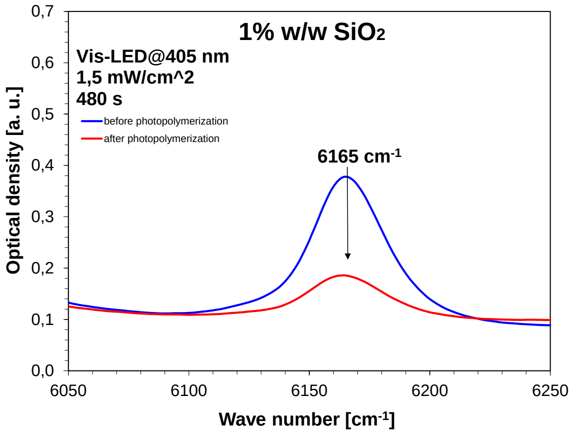

Figure S70. FT-IR spectra recorded before and after photopolymerization of nanocomposition polymerizing according to radical mechanism with 1% wt. SiO_2 for a 1,4 mm thick layer of the sample.

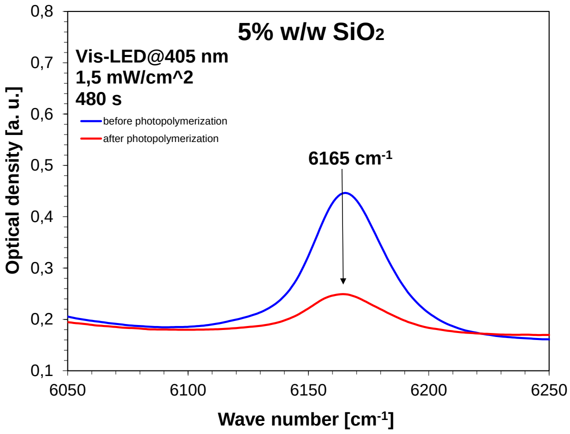

Figure S71. FT-IR spectra recorded before and after photopolymerization of nanocomposition polymerizing according to radical mechanism with 5% wt. SiO_2 for a 1,4 mm thick layer of the sample.

Changes in FT-IR spectra showing band decay at 6165 cm^{-1} wavenumber monitored for a cationic polymerizing composition for a test layer thickness of 1,4 mm.

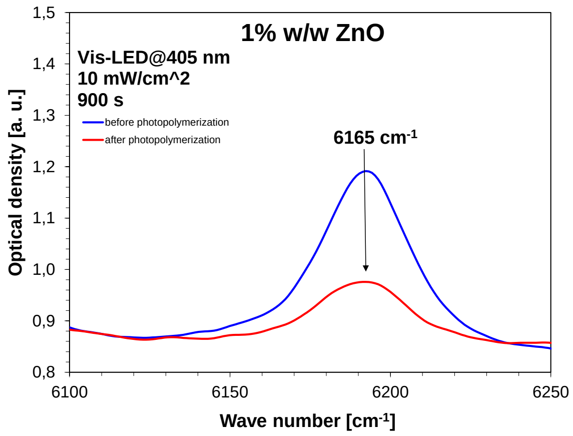

Figure S72. FT-IR spectra recorded before and after photopolymerization of nanocomposition polymerizing according to cationic mechanism with 1% wt. ZnO for a 1,4 mm thick layer of the sample.

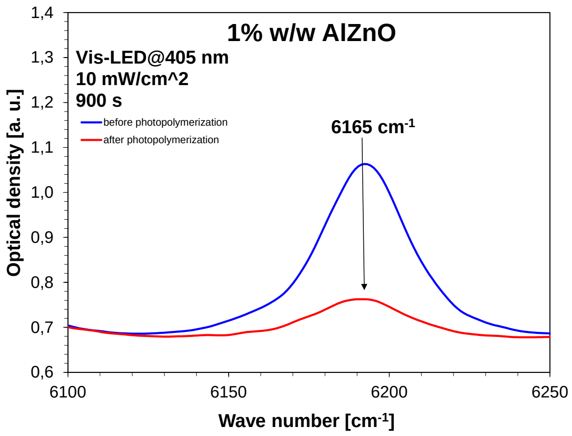

Figure S73. FT-IR spectra recorded before and after photopolymerization of nanocomposition polymerizing according to cationic mechanism with 1% wt. AlZnO for a 1,4 mm thick layer of the sample.

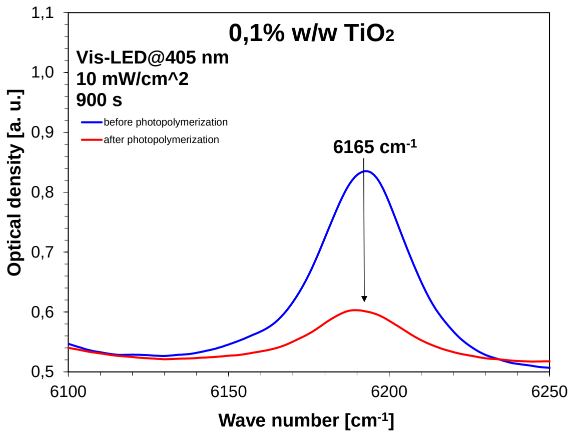

Figure S74. FT-IR spectra recorded before and after photopolymerization of nanocomposition polymerizing according to cationic mechanism with 0,1% wt. TiO_2 for a 1,4 mm thick layer of the sample.

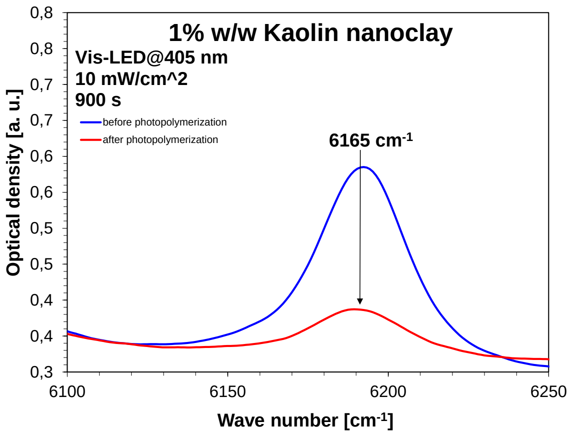

Figure S75. FT-IR spectra recorded before and after photopolymerization of nanocomposition polymerizing according to cationic mechanism with 1% wt. Kaolin nanoclay for a 1,4 mm thick layer of the sample.

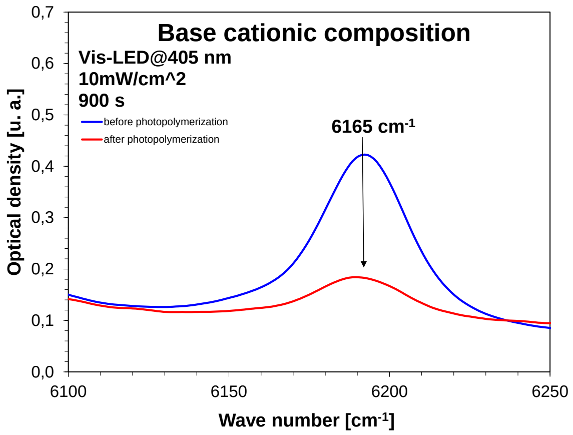

Figure S76. FT-IR spectra recorded before and after photopolymerization of nanocomposition polymerizing according to cationic mechanism for a 1,4 mm thick layer of the sample.

Cube models made using 3D printing from selected radical nanocompositions.

The photos in the green frame were taken with a standard camera.
The photos in the red frame were taken with an Olympus DSX1000 digital microscope.

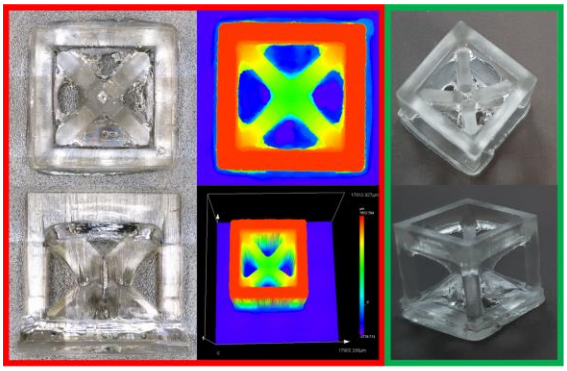

Figure S77. Printed from a base radical-reactive composition.

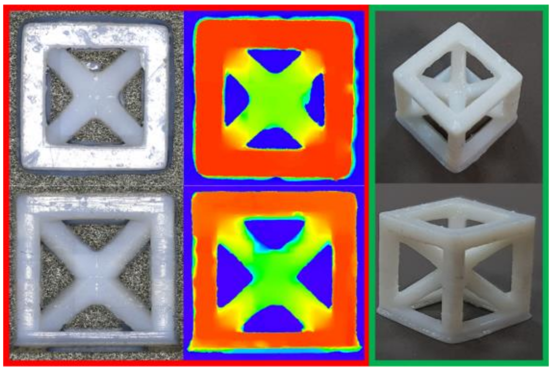

Figure S78. Printed from a radical-reactive composition with 5% wt. ZnO.

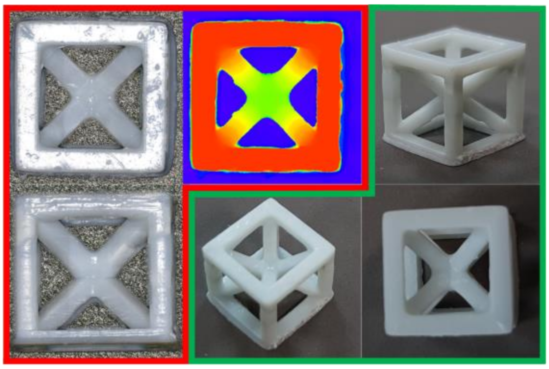

Figure S79. Printed from a radical-reactive composition with 5% wt. AlZnO.

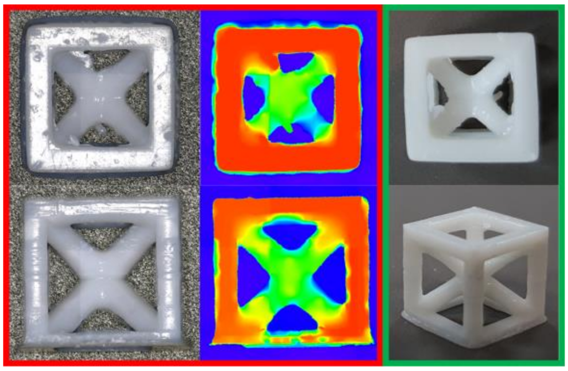

Figure S80. Printed from a radical-reactive composition with 5% wt. ZrO_2.

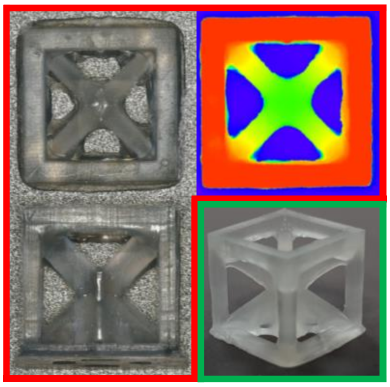

Figure S81. Printed from a radical-reactive composition with 5% wt. Kaolin nanoclay.

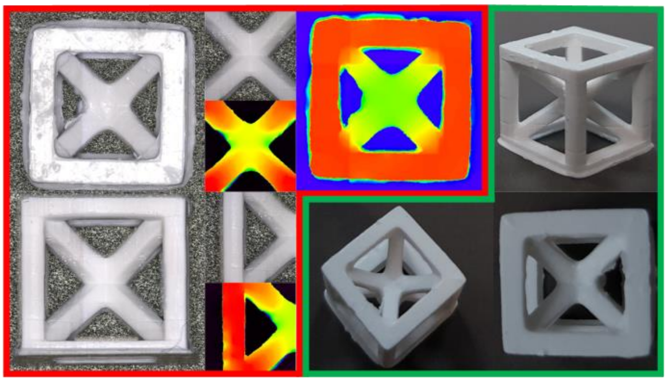

Figure S82. Printed from a radical-reactive composition with 5% wt. TiO_2.

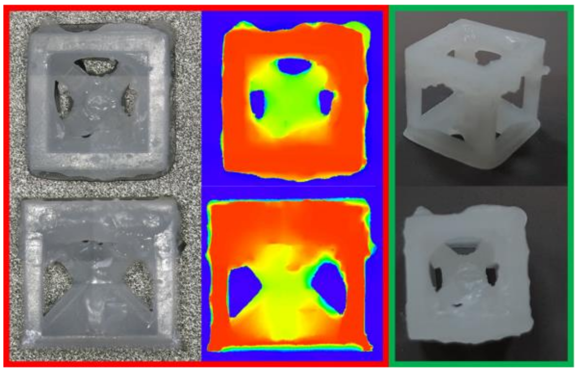

Figure S83. Printed from a radical-reactive composition with 5% wt. Al_2O_3.

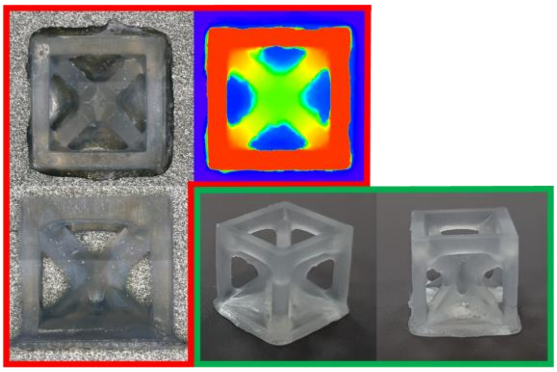

Figure S84. Printed from a radical-reactive composition with 5% wt. SiO_2.

Visualization of the roughness and waviness of the test area of the 3D prints.

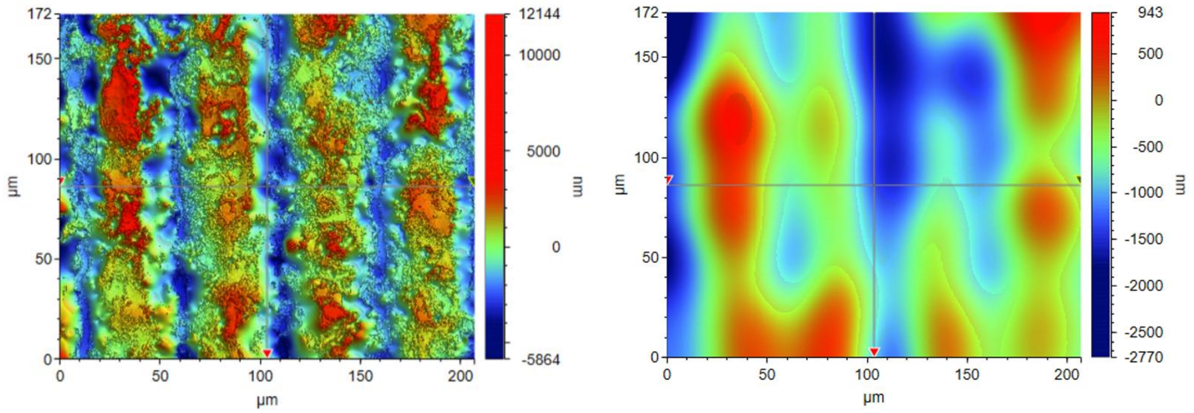

(a) **(b)**

Figure S85. Visualization of the roughness (a) and waviness (b) of the test area of the print from the nanocomposition containing 5% by weight Kaolin nanoclay. The tests were done in alignment A according to Figure 3.

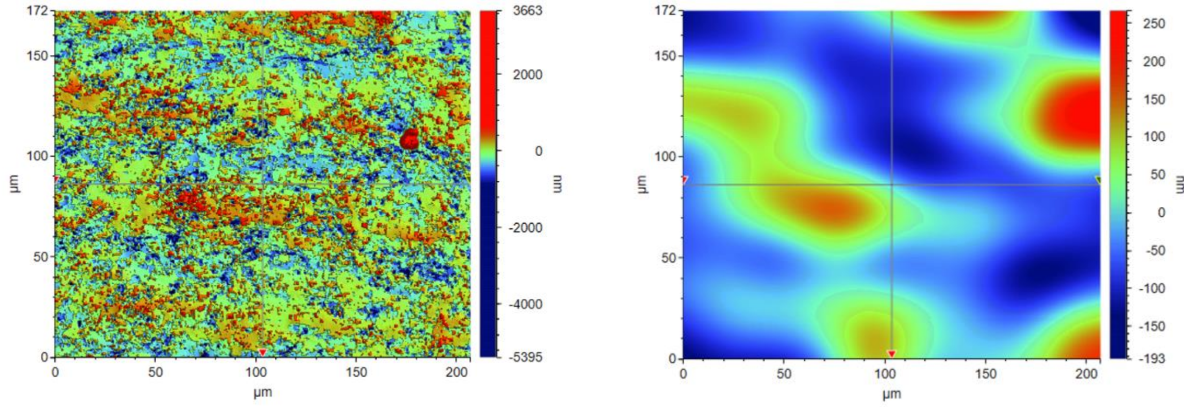

(a) **(b)**

Figure S86. Visualization of the roughness (a) and waviness (b) of the test area of the print from the nanocomposition containing 5% by weight TiO_2. The tests were done in alignment B according to Figure 3.

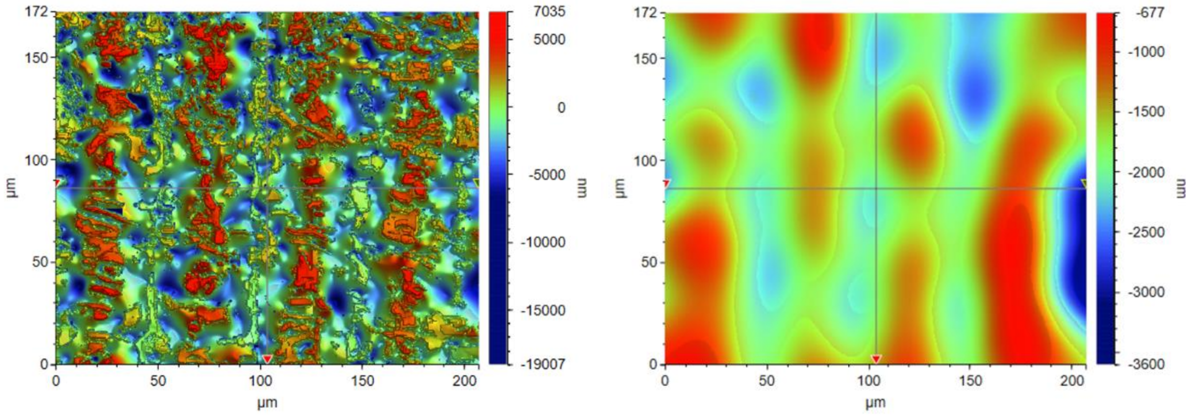

(a) **(b)**

Figure S87. Visualization of the roughness (a) and waviness (b) of the test area of the print from the radical base composition. The tests were done in alignment A according to Figure 3.

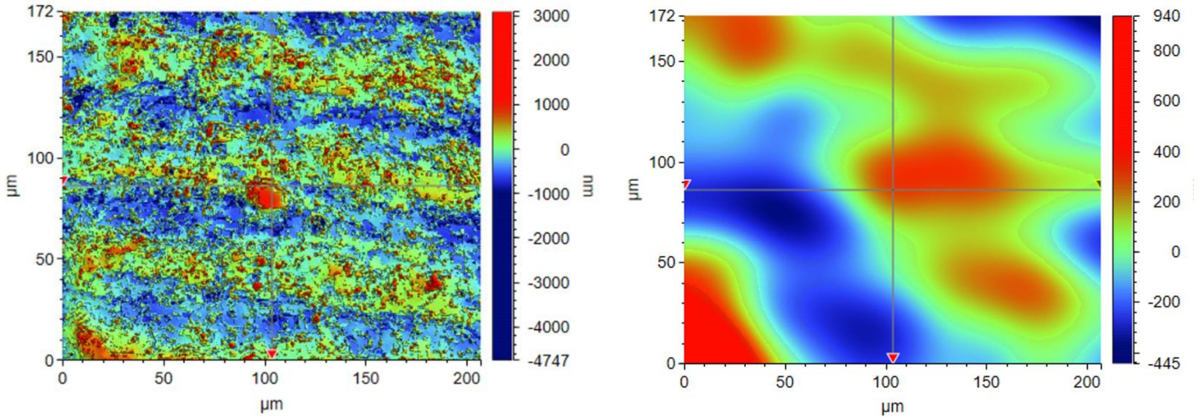

(a) **(b)**

Figure S88. Visualization of the roughness (a) and waviness (b) of the test area of the print from the nanocomposition containing 5% by weight ZnO. The tests were done in alignment B according to Figure 3.

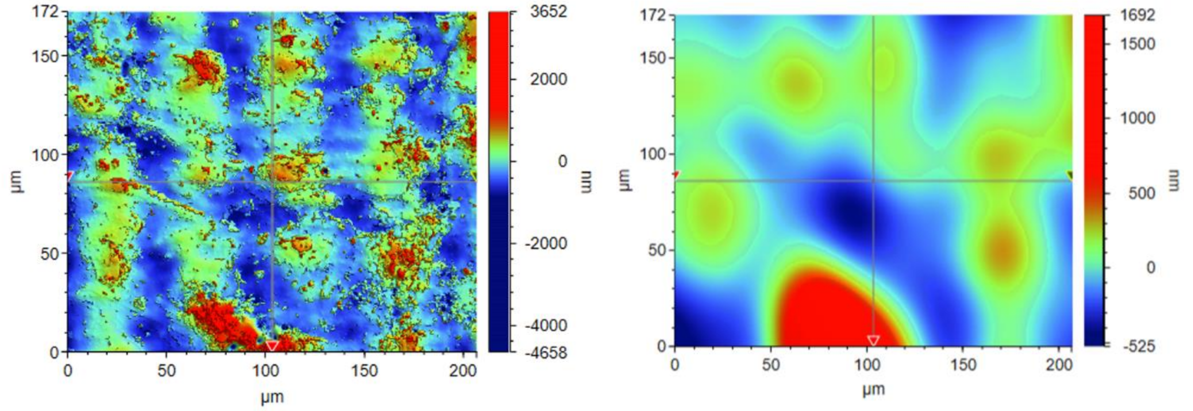

(a) **(b)**

Figure S89. Visualization of the roughness (a) and waviness (b) of the test area of the print from the nanocomposition containing 5% by weight ZrO_2. The tests were done in alignment A according to Figure 3.

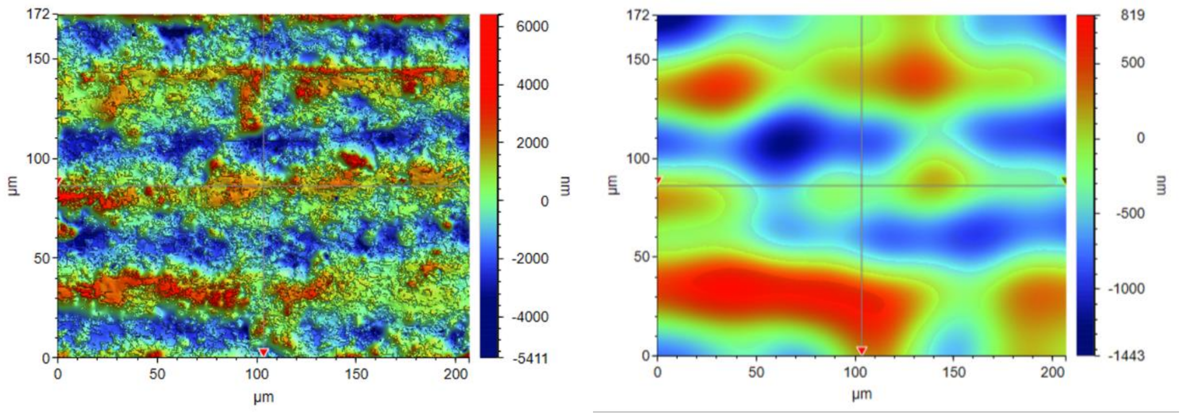

(a) **(b)**

Figure S90. Visualization of the roughness (a) and waviness (b) of the test area of the print from the nanocomposition containing 5% by weight Al_2O_3. The tests were done in alignment B according to Figure 3.

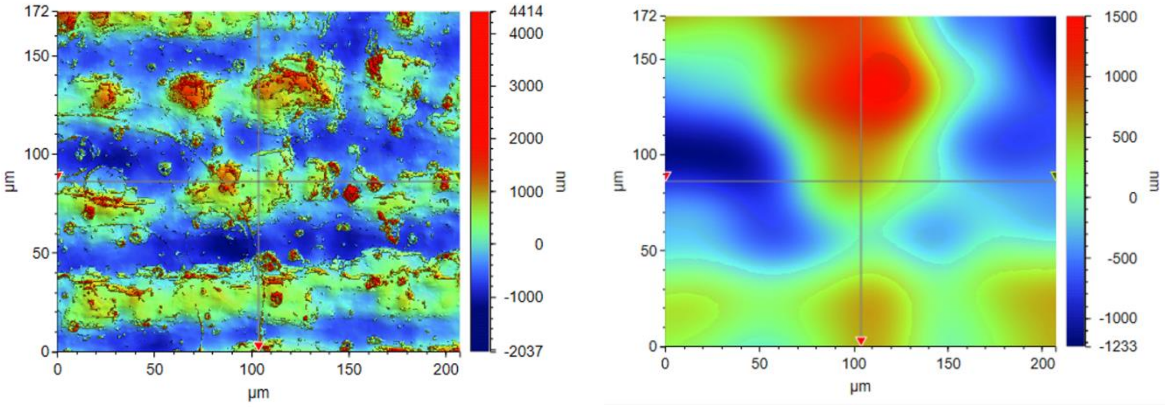

(a) **(b)**

Figure S91. Visualization of the roughness (a) and waviness (b) of the test area of the print from the nanocomposition containing 5% by weight AlZnO. The tests were done in alignment B according to Figure 3.

Analysis of the height of the tested surface of the 3D prints.

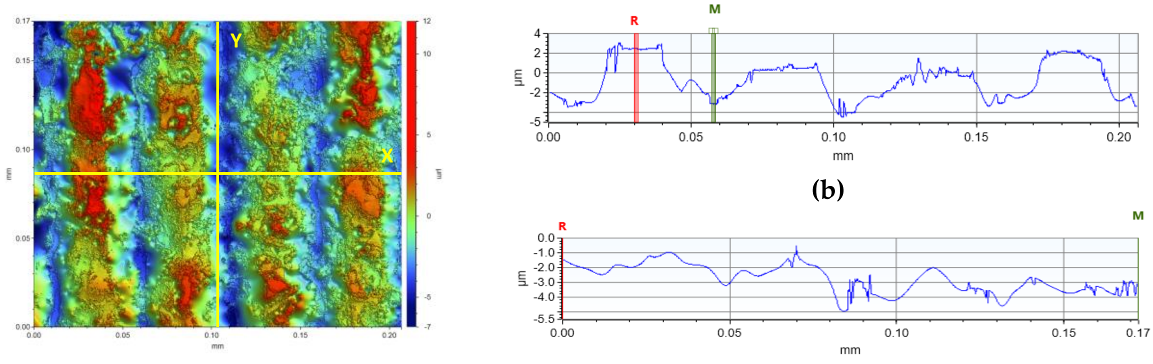

(a) **(c)**

Figure S92. Analysis of the height of the tested surface of the 3D print (**a**) from the polymer nanocomposite containing 5% by weight of kaolin nanoclay in the **(b)** X-axis (ΔX=0,0283 mm, ΔZ= -5,5671 µm) and in the **(c)** Y-axis (ΔX=0,1722 mm, ΔZ= -1,9012 µm).

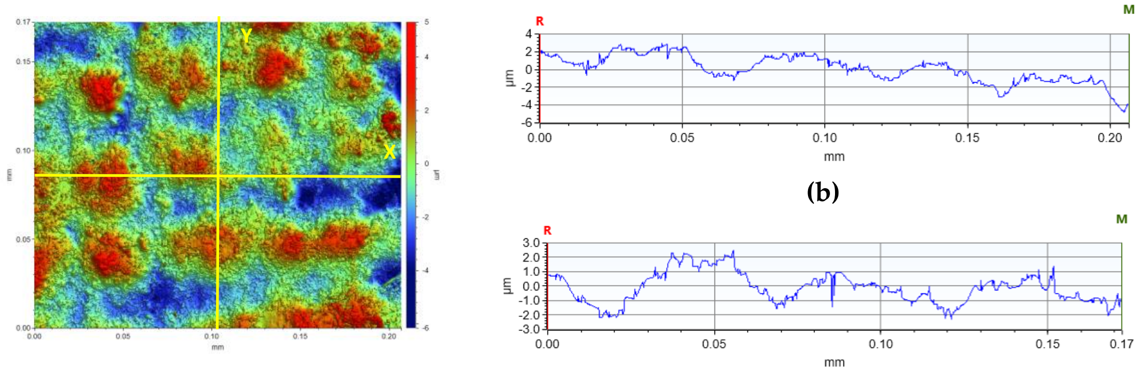

(a) (c)

Figure S93. Analysis of the height of the tested surface of the 3D print (**a**) from the polymer nanocomposite containing 5% by weight of SiO_2 in the **(b)** X-axis (ΔX=0,2067 mm, ΔZ= -5,7650 µm) and in the **(c)** Y-axis (ΔX=0,1722 mm, ΔZ= -1,4788 µm).

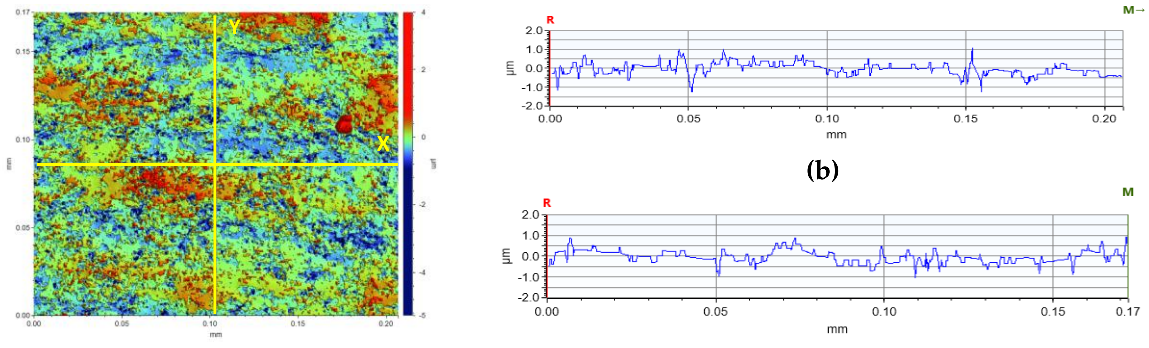

(a) (c)

Figure S94. Analysis of the height of the tested surface of the 3D print (**a**) from the polymer nanocomposite containing 5% by weight of TiO_2 in the **(b)** X-axis (ΔX=0,2067 mm, ΔZ= -0,1486 µm) and in the **(c)** Y-axis (ΔX=0,1722 mm, ΔZ= 0,8160 µm).

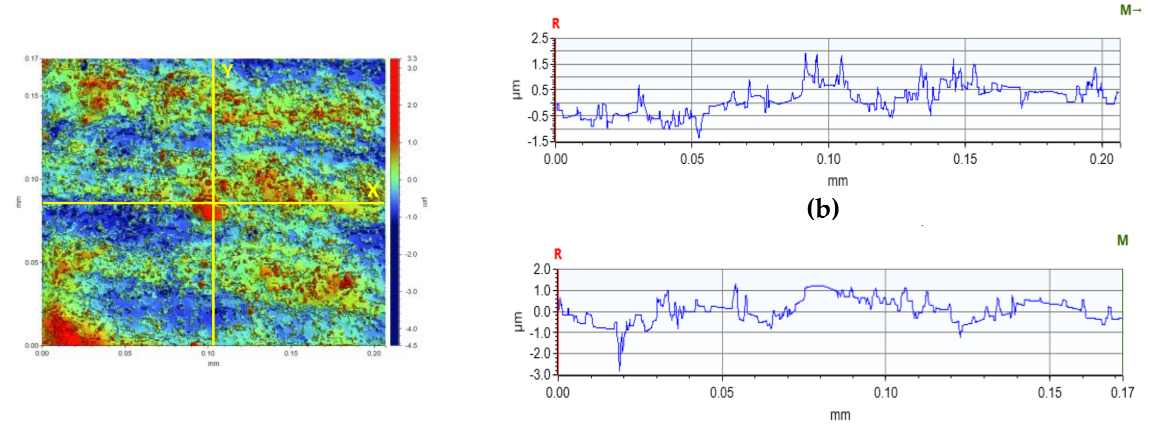

(a) (c)

Figure S95. Analysis of the height of the tested surface of the 3D print (**a**) from the polymer nanocomposite containing 5% by weight of ZnO in the **(b)** X-axis (ΔX=0,2067 mm, ΔZ= 0,9929 µm) and in the **(c)** Y-axis (ΔX=0,1722 mm, ΔZ= -0,2589 µm).

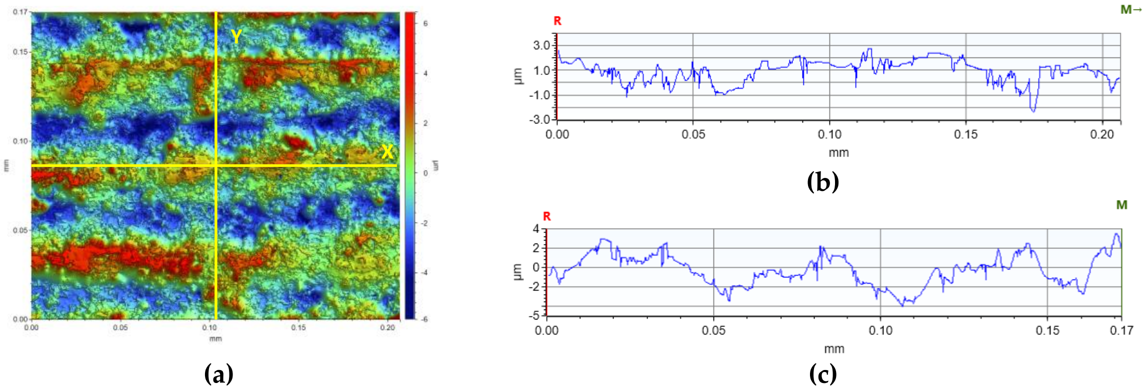

(a) **(b)** **(c)**

Figure S96. Analysis of the height of the tested surface of the 3D print (**a**) from the polymer nanocomposite containing 5% by weight of Al_2O_3 in the **(b)** X-axis (ΔX=0,2067 mm, ΔZ= -2,3823 µm) and in the **(c)** Y-axis (ΔX=0,1722 mm, ΔZ= 3,2009 µm).

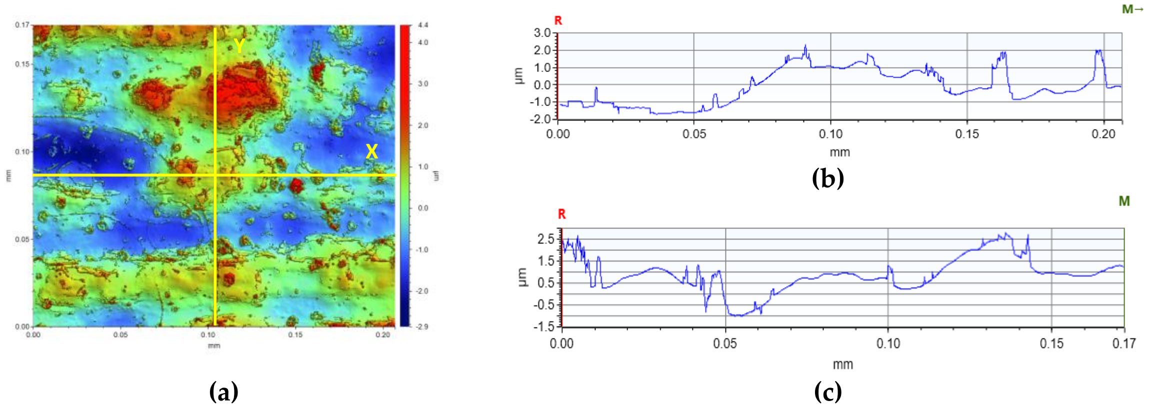

(a) **(b)** **(c)**

Figure S97. Analysis of the height of the tested surface of the 3D print (**a**) from the polymer nanocomposite containing 5% by weight of AlZnO in the **(b)** X-axis (ΔX=0,2067 mm, ΔZ= 1,0202 µm) and in the **(c)** Y-axis (ΔX=0,1722 mm, ΔZ= -0,7574 µm).

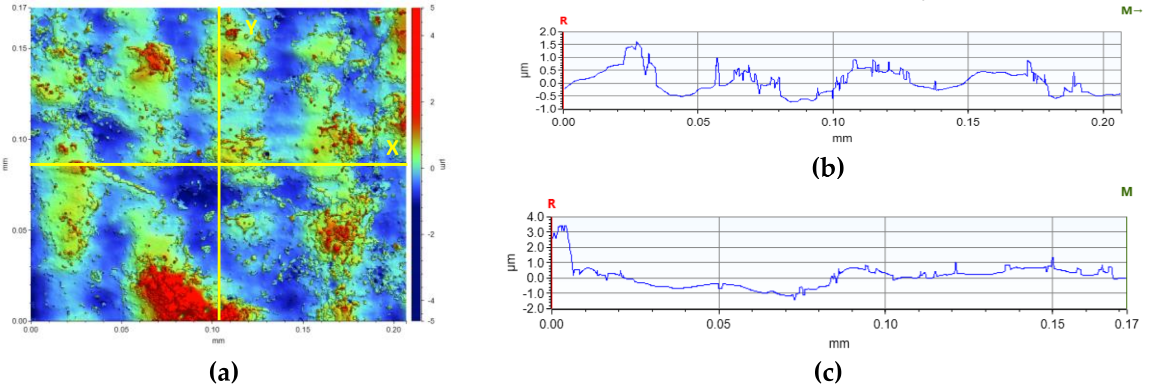

(a) **(b)** **(c)**

Figure S98. Analysis of the height of the tested surface of the 3D print (**a**) from the polymer nanocomposite containing 5% by weight of ZrO_2 in the **(b)** X-axis (ΔX=0,2067 mm, ΔZ= -0,1296 µm) and in the **(c)** Y-axis (ΔX=0,1722 mm, ΔZ= -2,8353 µm).
